# ADA2 Forms Nuclear Condensates with GCN5 and ATP‐Citrate Lyase (ACL) to Modulate H3K9 Acetylation at Genes Functioning in Rice Meristems

**DOI:** 10.1002/advs.202513169

**Published:** 2025-11-12

**Authors:** Yaping Yue, Tingting Lu, Xiaoyuan Guo, Biao Liu, Shiteng Lv, Heba A. M. Abdalla, Xuelei Lai, Ruihui Zhang, Jianpin Guo, Yu Zhao, Dao‐Xiu Zhou, Shaoli Zhou

**Affiliations:** ^1^ National Key Laboratory of Crop Genetic Improvement Hubei Hongshan Laboratory Huazhong Agricultural University Wuhan China; ^2^ Department of Botany Agriculture and Biological Research Institute National Research Centre Dokki Giza 12422 Egypt; ^3^ Institute of Plant Science Paris‐Saclay (IPS2) CNRS INRA University Paris‐Saclay Orsay 91405 France

**Keywords:** rice meristem, phase separation, GAA complex, ATP‐citrate lyase, H3K9 acetylation

## Abstract

SAGA (Spt‐Ada‐Gcn5 acetyltransferase) is a highly conserved histone acetyltransferase (HAT) complex in eukaryotes, playing a crucial role in regulating gene transcription during development. The complex consists of two core components: GCN5, the HAT subunit, and ADA2, which primarily functions as an adaptor and enhances the complex HAT activity. Beyond its well‐established roles, it is discovered in this work that ADA2 in rice possesses a broadly existing intrinsically disordered region (IDR) that directs the formation of nuclear condensates. Moreover, ADA2 is found to interact with a subunit of ATP‐citrate lyase (ACL), an enzyme that produces acetyl‐CoA, leading to the formation of a GCN5‐ADA2‐ACL (GAA) complex. ADA2 promotes the condensation of both GCN5 and ACL in vivo. Within these condensates, ACL contributes to the production and enrichment of acetyl‐CoA, thereby promoting histone acetylation. Genetic evidence showed that knock‐out or suppression of these genes led to similarly diminished root meristem zone sizes and reduced branch primordia numbers, accompanied by significant reductions in genomic H3K9 acetylation and transcriptional attenuation of essential genes for meristem function. In summary, the findings unveil a novel mechanism of HAT action by forming phase separation to enrich acetyl‐CoA within nuclear puncta, facilitating histone acetylation at target genes essential for meristem development.

## Graphical abstract

This study unveils a novel GAA (GCN5‐ADA2‐ACL) complex that forms nuclear condensates to boost local acetyl‐CoA, facilitating histone acetylation and gene transcription in rice meristems. The research underscores the role of ADA2's disordered region in complex condensation and ACL's role in acetyl‐CoA production. The findings offer key insights into the mechanisms by which the histone acetylation complex works in plant meristems.

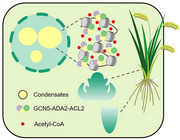

## 1 Introduction

Histone modifications, such as lysine acetylation, play crucial roles in regulating chromatin status and gene transcription. Histone acetylation consumes acetyl coenzyme A (acetyl‐CoA) and is deposited by the actions of histone acetyltransferases (HATs). The SAGA complex is the earliest identified histone acetyltransferase complex in eukaryotes.^[^ ^1^, ^2^, ^3^, ^4^ ^]^ Among its components, GCN5 acts as the catalytic core, with ADA2 serving as an adaptor protein, providing a scaffold for recruiting other functional proteins and assisting GCN5 in accessing target amino residues to facilitate catalytic activity.^[^ ^5^, ^6^, ^7^ ^]^ The interaction between GCN5 and ADA2 is detected in diverse eukaryotes, including rice.^[^ ^8^, ^9^, ^10^ ^]^ It is shown that this complex plays an important role in promoting cell proliferation and modulating root development in rice.^[^ ^11^ ^]^ A recent study indicates that the dynamics of lysine acetylation of ADA2 in response to stress or low cellular acetyl‐CoA levels regulate GCN5 HAT activity, thereby conferring stress tolerance in rice.^[^ ^12^ ^]^

Mounting evidence emphasizes the functional significance of protein condensation in regulating plant development and adaptation to the ambient environment.^[^ ^13^, ^14^, ^15^ ^]^ For instance, the co‐liquid liquid phase separation (LLPS) of FLL2 and Flowering Control Locus A (FCA) is required for alternative polyadenylation and transcription of the *FLOWERING LOCUS C (FLC)*, a major flowering regulator in *Arabidopsis thaliana*.^[^ ^16^ ^]^ The ELF3 protein, containing a poly‐Q repeat, was found to undergo phase transition in response to temperature alterations, contributing to thermo‐morphogenesis in plants. Two RNA‐binding proteins, RBGD2 and RBGD4 (RNA‐binding glycine‐rich D2 and D4), were identified to undergo LLPS in vitro and condense into heat‐induced stress granules in vivo via tyrosine residue array (TRA). A mutation in TRA abolished the condensation of RBGD and impaired plant heat resistance.^[^ ^17^ ^]^ The plant‐specific histone methyltransferase SUVR2 was shown to undergo LLPS to promote DNA repair in barrel clover (*Medicago truncatula*).^[^ ^18^ ^]^ Whether phase separation, a widely observed phenomenon, plays a role in regulating histone acetylation in the nucleus remains unknown.

Increasing studies indicate that metabolic enzymes are directly involved in genomic transcription regulation. For instance, α‐ketoglutarate dehydrogenase (KGDH) responds to light and translocates between the nucleus and mitochondria. Nuclear‐localized KGDH interacts with various histone demethylase family members (JMJs) and catalyzes the oxidative decarboxylation of α‐KG, which is necessary for histone demethylation reactions, thereby affecting genomic histone methylation and altering gene transcription in *Arabidopsis thaliana*.^[^ ^19^ ^]^ Glyceraldehyde‐3‐phosphate dehydrogenase (GAPDH) was found to act as a transcription factor in accelerating the expression of downstream glycolytic genes in rice. Its transcriptional activity is regulated by the OsSRT1‐dependent lysine deacetylation.^[^ ^20^ ^]^ Additionally, the ATP‐citrate lyase (ACL), a key metabolic enzyme catalyzing the conversion of citrate and coenzyme A (CoA) into acetyl‐CoA, ADP, and phosphate, was found to directly interact with histone acetyltransferases HAG704 in regulating histone H4K5 acetylation in rice.^[^ ^21^ ^]^ Knockout of these two genes impacts both impact nuclear division and the development of rice endosperm.^[^ ^21^ ^]^

In our work, we identified that ADA2 recruits both GCN5 and ACL to form the GCN5‐ADA2‐ACL (GAA) complex. Within this complex, ADA2 functions to mediate the condensation of the GAA complex in the nucleus through its intrinsically disordered region, accompanied by ACL, which enriches acetyl‐CoA in the condensates to stimulate GCN5 HAT activity. Mutations in the three components all lead to a decrease in genomic H3K9 acetylation, misregulation of a set of functional genes, and impaired root and inflorescence development. Our work unravels a novel mode of action of the histone acetyltransferase complex by forming nuclear condensates with ATP‐citrate lyase to locally enrich acetyl‐CoA for chromatin regulation of gene transcription.

## 2 Results

### 2.1 GCN5‐ADA2‐ACLA2 forms a Complex in Rice

**Figure 1:**
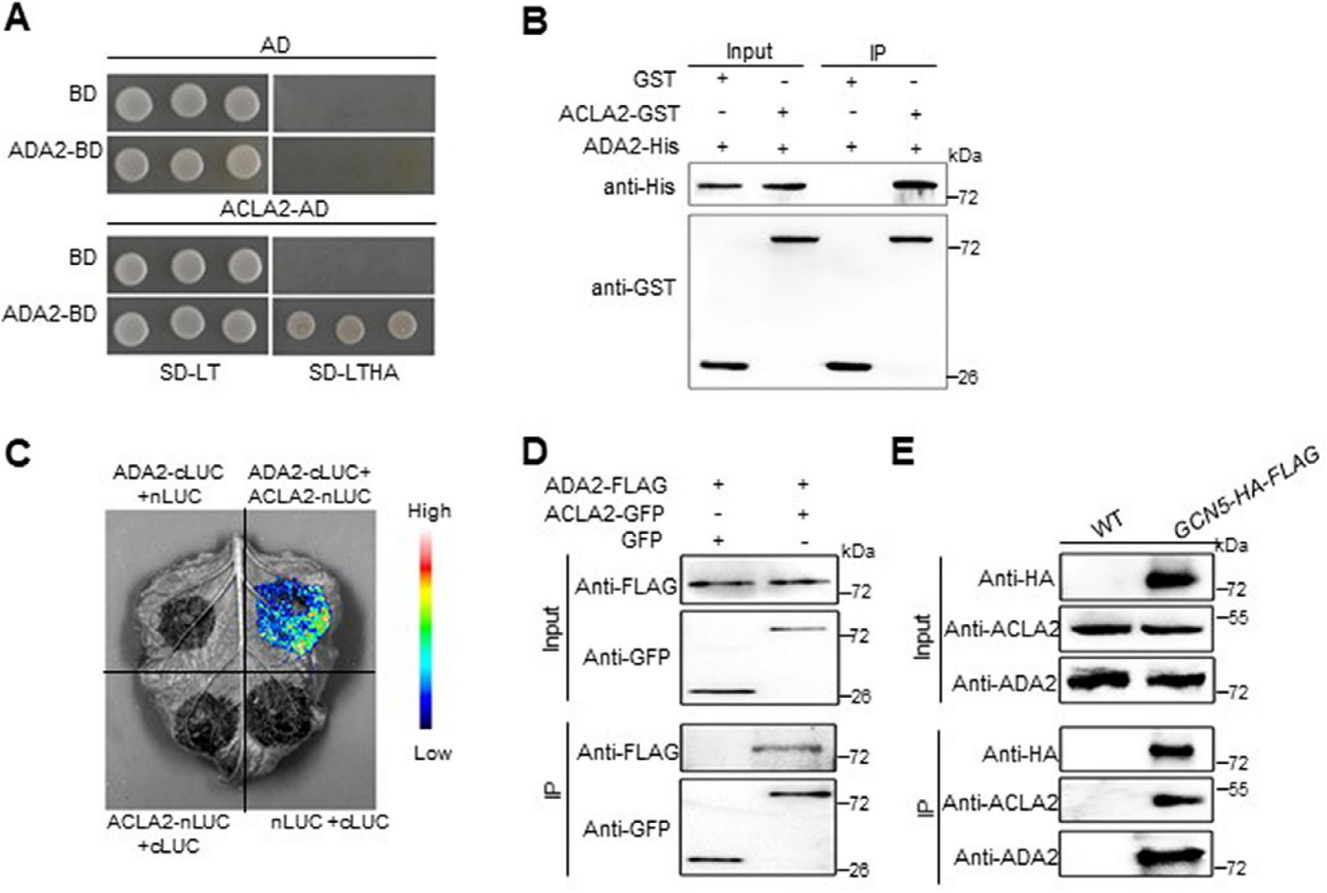
GCN5‐ADA2‐ACLA2 works as a complex (GAA complex) in rice. A) Yeast two‐hybrid assays of ADA2 and ACLA2 in yeast cells. Full‐length cDNAs of *ADA2* and *ACLA2* were cloned into BD (the bait plasmid pGBKT7) or AD (the prey plasmid pGADT7). Yeast cells transformed with the indicated plasmid combinations were grown on control SD‐LT medium and selective SD‐LTHA medium. B) Pull‐down assays of ADA2 and ACLA2. ADA2‐His fusion protein was incubated with either GST or ACLA2‐GST. Commercial GST beads were utilized to pull down the GST or GST‐tagged proteins, as well as their potential interacting proteins. C) Split‐luciferase complementation assays (SLC) showing the interaction between ADA2 and ACLA2 in the leaves of tobacco. D) Co‐immunoprecipitation assays of ADA2 and ACLA2 in the rice protoplast system. The *35S::ADA2‐FLAG* construct was co‐transfected into rice protoplasts with either *35S::ACLA2‐GFP* or *GFP* alone. After overnight incubation, the protein complex was immunoprecipitated using commercial anti‐FLAG beads. The IP products were analyzed by immunoblotting with anti‐FLAG and anti‐GFP antibodies. E) Co‐immunoprecipitation assays showing that GCN5‐ADA2‐ACLA2 form a complex in vivo. The cell lysis products from either wild‐type (WT) or *GCN5* overexpression plants were immunoprecipitated with commercial anti‐FLAG beads. Samples of input and immunoprecipitation products were analyzed by immunoblots with anti‐HA, anti‐ACLA2, and anti‐ADA2 antibodies.

ACLs in plants are split into two subunits, ACLA and ACLB, with three homologs for ACLA and one copy for ACLB in the rice genome.^[^ ^21^, ^22^, ^23^ ^]^ In our previous work, we observed that ADA2 interacts with ACLA2 in yeast.^[^ ^21^ ^]^ To confirm the interaction between these two proteins and to investigate the potential ternary complex (GCN5‐ADA2‐ACLA2), we conducted a series of experiments to validate the interactions both in vitro and in vivo. As shown in **Figure** **1**A, ADA2 interacts with ACLA2 in the yeast two‐hybrid system, as previously reported. In addition to this, we expressed FLAG‐fused proteins in *Escherichia coli* system. The pull‐down assay revealed that ADA2‐His interacts with ACLA2‐GST, with no detectable signal in the GST control (Figure 1B). The split‐luciferase complementation (SLC) assay showed that ADA2 interacts with ACLA2 in tobacco cells (Figure 1C). To study the in vivo interaction, we expressed the proteins in rice protoplasts. Cells co‐expressing ADA2‐FLAG with either ACLA2‐GFP or GFP were lysed and immunoprecipitated using commercial anti‐GFP beads. As shown in Figure 1D, ADA2 was successfully pulled down only by ACLA2‐GFP, not by GFP. These data collectively demonstrate that ADA2 and ACLA2 indeed interact with each other both in vitro and in vivo.

Given that ADA2 serves as the core component of the SAGA complex and generally acts as a scaffold for GCN5 in the nucleus, we hypothesized that GCN5‐ADA2‐ACLA2 forms a complex in rice. To test this hypothesis, we performed Co‐Immunoprecipitation (Co‐IP) in both the GCN5‐HA‐FLAG transgenic line and wild‐type plants.^[^ ^11^ ^]^ By precipitating with commercial anti‐FLAG beads, ADA2 and ACLA2 were enriched in the GCN5‐HA‐FLAG Co‐IP product compared to the wild type (Figure 1E), suggesting that the GCN5‐ADA2‐ACLA2 (GAA) complex indeed functions in rice cells.

### 2.2 GCN5, ADA2, and ACLA2 Simultaneously Affect Meristem Size and Cell Division in Rice

**Figure 2:**
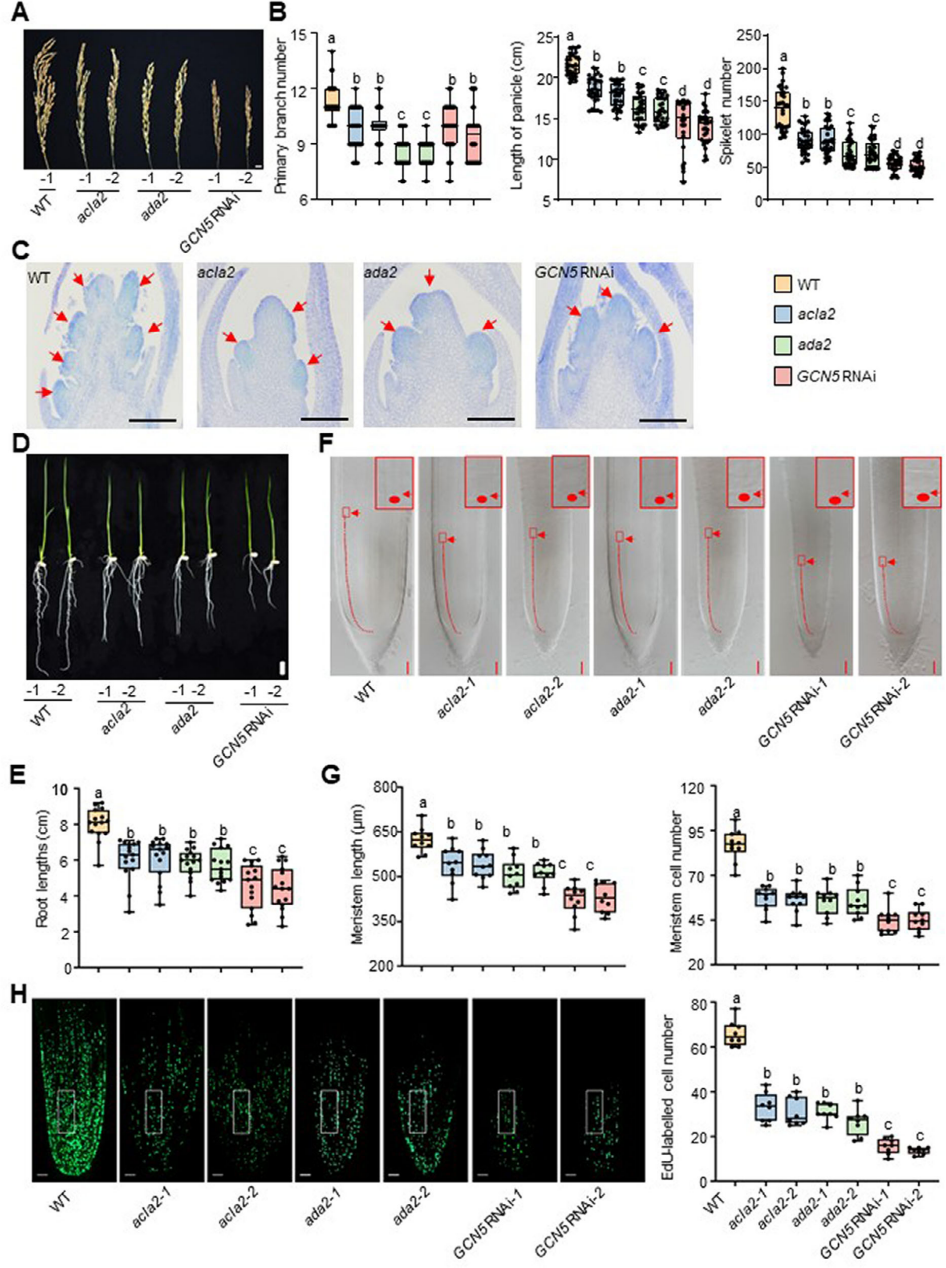
GCN5, ADA2, and ACLA2 co‐regulate the development of rice root and inflorescence meristems. A) Images show the mature panicle of wild type (WT), *acla2*, *ada2*, and *GCN5* RNAi. Bar = 1 cm. B) Boxplots illustrate the statistics of primary branch number, length of panicle, and spikelet number in the indicated backgrounds (*n* = 30 for each). C) Longitudinal sections of inflorescence meristems from the indicated genotypes. Red arrows indicate the primary branch primordia. Bars = 100 µm. D, E) Image and boxplots showing the root phenotype and their statistics of the indicated background materials at their 7‐day‐old age. Bars = 1 cm, *n* = 15 for each. F) Median longitudinal sections of wild type (WT), *acla2*, *ada2*, and *GCN5* RNAi root tips. Arrows indicate the end of the meristem (from the quiescent center to the transition zone). Red boxes are the enlarged images for the end of the meristem zones. Bars = 50 µm. G) Statistical analysis for meristem lengths (left panel) and cell numbers within the meristem zone (right panel) in the indicated backgrounds (n = 10 for each). H) Left panel, EdU‐labeled cells in 4‐day‐old seedling root meristem*s* of wild type (WT), *acla2, ada2, and GCN5* RNAi plants. Bars = 50 µm. Right panel, boxplots show the numbers of EdU‐labeled cells in an arbitrary area of the root tip (white box). *n* = 9, EdU, 5‐ethynyl‐2′‐deoxyuridine. For all statistics, boxplots show the 25th and 75th percentiles (box), median, and highest and lowest values. Error bars represent the means ± SD of three independent biological replicates. The different significances were calculated using one‐way ANOVA with Tukey's multiple comparison tests. Different letters on top of the bars indicate a significant difference (*p‐*value < 0.05), and the same letters on top of bars indicate no significant difference.

To investigate whether the three genes are involved in the same regulatory process in rice, we inspected the phenotypes of both the vegetative and reproductive stages. For the reproductive stage, panicles were found to be significantly diminished in *GCN5* RNAi, *ada2*, and *acla2* mutants compared to the wild type (**Figure** **2**A). Further observation revealed that the average primary branch number, spike length, and mean spikelet per panicle were all significantly reduced in the three mutants/RNAi backgrounds (Figure 2B). In situ hybridization experiments showed *GCN5*, *ADA2*, and *ACLA2* were significantly expressed at branch meristems (Figure S1A–F, Supporting Information), suggesting their functions in the indicated organogenesis process. Histological assays further revealed a decrease in the number of primary branch primordia in *GCN5* RNAi, *ada2*, and *acla2* mutants relative to wild type (Figure 2C; Figure S1G, Supporting Information), consistent with observations of mature panicle phenotypes. Collectively, these data suggest that the GAA complex plays a role in inflorescence development in rice.

As reported,^[^ ^11^, ^21^ ^]^ *GCN5* RNAi, *ada2*, and *acla2* mutants similarly exhibited a significant reduction in root length compared to the wild type (Figure 2D,E). Examination of the meristem revealed substantial decreases in cell numbers along the longitudinal direction in the indicated mutants and transgenic lines relative to the wild type (see methods) (Figure 2F,G). Staining with 5‐ethynyl‐20‐deoxyuridine (Edu), which marks the newly synthesized DNA strand during replication, showed a significantly decreased cell ratio undergoing DNA replication in the three indicated materials compared to the wild type (Figure 2H). These results suggest that cell division activity is significantly affected by the absence of any component of the GAA complex.

### 2.3 ADA2 Condenses in the Nucleus

**Figure 3:**
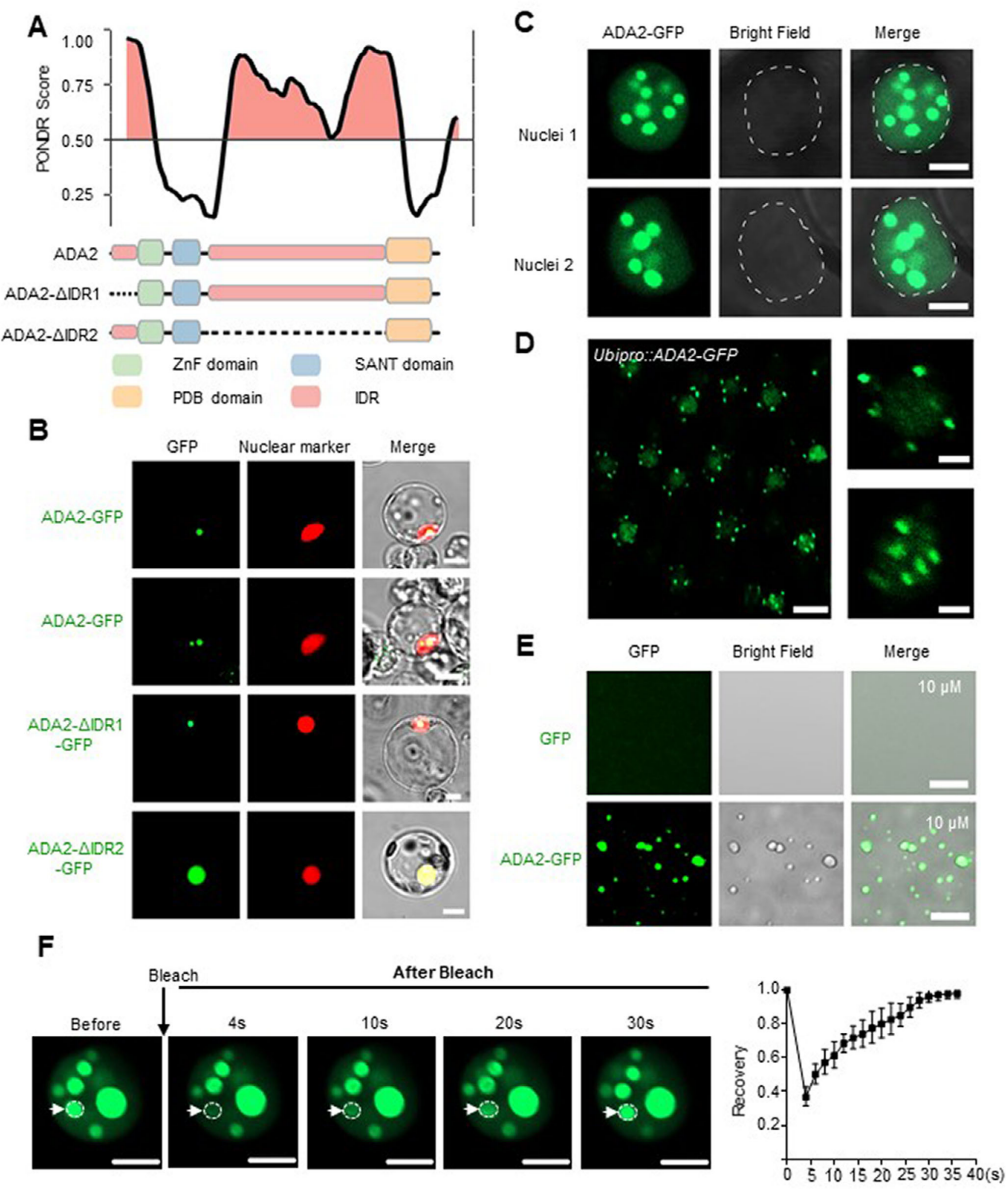
ADA2 phase separated in vitro and in vivo. A) Top panel, intrinsically disordered region (IDR) prediction for ADA2 by PONDR database (http://pondr.com/). Regions with an average strength (PONDR score) ≥ 0.5 were considered to be disordered. Bottom panel, schematic diagrams showing the domain structures of the ADA2 protein and its two truncations. ZnF, zinc finger. SANT, SWI3‐ADA2‐N‐COR‐TFIIIB domain. B) Transient protein expression in the rice protoplast system shows that ADA2‐GFP, but not ADA2‐ΔIDR2‐GFP (deletion of IDR2), aggregated in the nucleus. SRT1‐RFP was used as the nuclear marker. Bars = 10 µm. C) ADA2‐GFP fusion protein aggregated in the nucleus of tobacco epidermal cells. Bars = 5 µm. D) Images showing the in vivo nuclear distribution of ADA2‐GFP. Root tips isolated from the *Ubipro::ADA2‐GFP* transgenic line were used for imaging. The scale bars in the left and right panels were 10 µm and 2 µm, respectively. E) In vitro phase separation assay for purified ADA2‐GFP protein. GFP protein was used as the negative control. Bars = 5 µm. F) Fluorescence recovery after photobleaching (FRAP) assay for ADA2‐GFP droplets in tobacco epidermal cells. Representative images before and after photobleaching are shown in the left panel. Bars = 5 µm. Quantification of the relative fluorescence recovery of ADA2‐GFP is illustrated in the right panel. Error bars represent the means ± SD from three independent biological replicates.

Investigation of the peptide characteristics revealed that ADA2 contains two intrinsically disordered regions (IDRs) in the N‐terminal and middle region between the functional domains (**Figure** **3**A). IDRs are known to be broadly involved in driving protein phase separation.^[^ ^24^, ^25^, ^26^ ^]^ These data implied that ADA2 might function as a phase‐separated protein in the nucleus. To validate this, we expressed ADA2 proteins fused with GFP in rice protoplasts and tobacco leaves and observed that ADA2 gathered and formed puncta in both nuclei (Figure 3B,C).

To determine which of the two IDRs is involved in the condensation of ADA2 in the nucleus, we created constructs with deletions of IDR1 and IDR2, respectively (Figure 3A,B). As shown in Figure 3B, the loss of IDR1 showed no impact on ADA2 condensate formation, whereas the loss of IDR2 abolished condensate formation of the protein in rice nuclei, suggesting that IDR2 plays a primary role in this process.

To further confirm the condensation behavior in vivo, we constructed transgenic lines: *Ubipro::ADA2‐GFP*, *ADA2pro::ADA2‐GFP*, and *ADA2pro::ADA2‐ΔIDR2‐Venus* (IDR2 deletion). Observations validated the aggregation state of ADA2 protein in rice root tip cells (Figure 3D; Figure S2A, Supporting Information). However, deletion of IDR2 destroyed protein aggregation in the cells (Figure S2B), consistent with the results of the transient protein expression assay.

Next, we investigated the phase separation behavior of ADA2 in vitro. The purified ADA2‐GFP formed droplets upon addition of the crowding agent polyethylene glycol 8000 (Figure 3E). In contrast, GFP alone failed to produce droplets. Fluorescence recovery after photobleaching (FRAP) assays showed that the GFP signal recovered rapidly after photobleaching in vivo (Figure 3F), indicating that ADA2 condensates are highly dynamic in cells.

To understand the driving force behind the phase separation behavior of ADA2 IDR, we examined the peptide composition and found that IDR2 was enriched in positively charged (K, R, and H) and negatively charged (D and E) amino acid residues relative to the proteome average levels (Figure S3A, Supporting Information). We therefore constructed two ADA2 variants by mutating these residues to Ala (KRH/A and DE/A, respectively) and examined their molecular behavior in the tobacco nucleus (Figure S3B,C, Supporting Information). As shown in Figure S3D (Supporting Information), the ADA2‐KRH/A variant completely diffused, while the ADA2‐DE variant formed smaller condensates in the nucleus. These suggest that the positively charged amino acid residues in the IDR are key for droplet formation of ADA2 protein, implying that electrostatic forces may play an important role in the phase separation behavior of ADA2.

ADA2 was reported to be modified by lysine acetylation.^[^ ^12^ ^]^ The introduction of acetyl groups is thought to alter protein charge,^[^ ^27^, ^28^, ^29^ ^]^ potentially influencing the phase separation of ADA2. To test this hypothesis, we mutated the seven lysine residues modified by acetylation into arginine (7KR) and glutamine (7KQ) (Figure S4A, Supporting Information). The 7KR mutation mimicked the non‐acetylated state of lysine, while the 7KQ mutation mimicked the acetylated state.^[^ ^12^, ^30^ ^]^ As shown in Figure S4B (Supporting Information), the puncta numbers and the nuclear area occupied by the condensates were significantly reduced in the 7KR mutant, whereas the condensate occupation area was markedly increased in 7KQ. These indicated that the acetylation of ADA2 positively impacts protein condensation.

### 2.4 Condensation of GCN5 and ACLA2 Depends on ADA2 in the Nucleus

**Figure 4:**
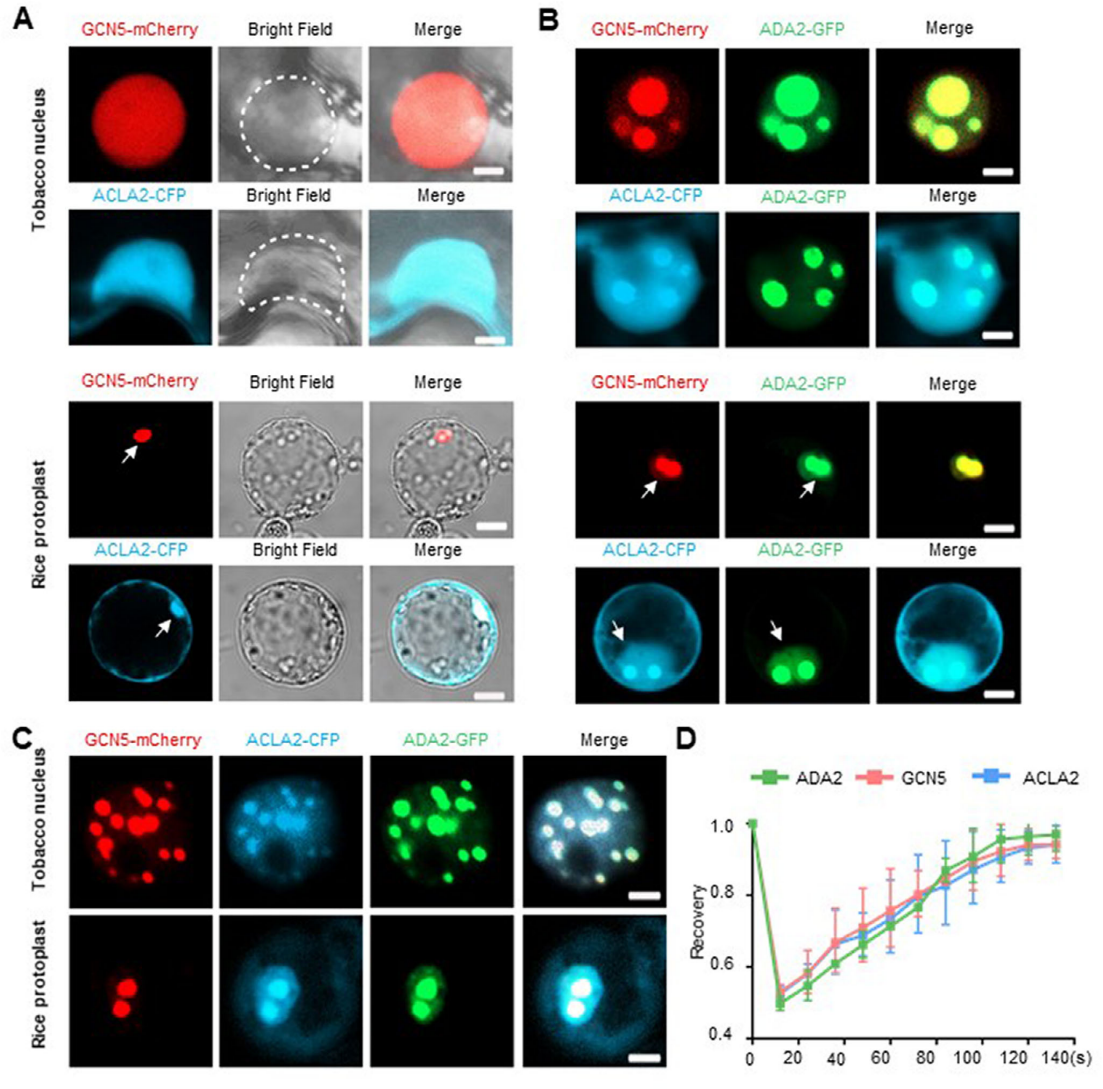
GCN5 and ACLA2 condensate formation is dependent on ADA2. A) Fluorescence images of GCN5‐mCherry and ACLA2‐CFP proteins in the nucleus of either tobacco epidermal cells (top panel) or rice protoplasts (bottom panel). Bars = 5 µm. B) Fluorescence images showing the nuclear distribution of GCN5‐mCherry and ACLA2‐CFP when co‐expressed with ADA2‐GFP in either tobacco nuclei (top panel) or rice protoplasts (bottom panel). ACLA2‐CFP is distributed in both the cytoplasm and nucleus, with ADA2‐GFP solely distributed within the nucleus. Arrows indicate the position of the nuclei. Bars = 5 µm. C) GCN5‐mCherry, ACLA2‐CFP, and ADA2‐GFP co‐condensate in the nucleus of tobacco cells (top panel) and rice protoplasts (bottom panel). Bars = 5 µm. D) FRAP assay of the GAA complex. Error bars represent the means ± SD from three independent biological replicates.

Given that GCN5, ADA2, and ACLA2 function as a complex in rice, we investigated whether GCN5 and ACLA2 also form condensates in the nucleus. To this end, GCN5‐mCherry and ACLA2‐CFP were expressed in tobacco leaves and rice protoplasts, respectively. As shown in **Figure** **4**A, GCN5 and ACLA2 were diffusely distributed throughout the nucleus, indicating that these two proteins do not spontaneously aggregate in the nucleus. However, when GCN5 or ACLA2 was co‐expressed with ADA2, noticeable condensates of GCN5 and ACLA2 formed (Figure 4B). Moreover, when GCN5, ADA2, and ACLA2 were expressed simultaneously in a single cell, they were coalesced into the same droplets (Figure 4C). The FRAP experiment showed that the GAA complex condensates inherit the dynamic characteristics of ADA2 droplets (Figure 4D; Figure S5, Supporting Information).

Additionally, we assessed the interaction between GCN5 (or ACLA2) and ADA2 variants containing mutations in charged amino acids. The results showed that the mutations of ADA2 did not affect their physical interaction with GCN5 or ACL2A (Figure S6A, Supporting Information). Consistent with the condensation abolition for ADA2 variants (Figure S3D, Supporting Information), GCN5 and ACLA2 failed to form puncta in the nucleus when they were co‐expressed with the ADA2‐KRH/A variant (Figure S6B, Supporting Information). These findings collectively demonstrate that the aggregation of the GAA complex in the nucleus is ADA2‐dependent.

### 2.5 ACL Accelerates Acetyl‐CoA Concentration in the Condensates

ATP‐Citrate lyase is the primary producer of acetyl‐CoA in cells, providing the acetyl source for histone acetylation reactions.^[^ ^31^, ^32^, ^33^ ^]^ The intact activity of ATP‐Citrate lyase relies on the association of ACLA and ACLB in plants.^[^ ^21^, ^32^, ^34^, ^35^ ^]^ To further confirm that all the essential components for citrate lyase activity are present within the GAA complex condenses, we tested the interaction between ACLA2 and ACLB. The results from yeast two‐hybridization and SLC assays in tobacco confirmed their interaction, similar to the interaction between ACLA1 and ACLB as previously reported^[^ ^36^ ^]^ (Figure S7A,B, Supporting Information). Sequentially, ADA2‐GFP and ACLB‐mCherry were produced in rice protoplast in the presence or absence of ACLA2‐CFP. As shown in Figure S7C,D (Supporting Information), ACLB was incorporated into ADA2 condensates only in the presence of ACLA2. Without ACLA2, ACLB was diffused in the nuclear space. These data indicate that the whole ATP‐Citrate lyase complex is co‐condensed within ADA2 droplets in the nucleus.

**Figure 5:**
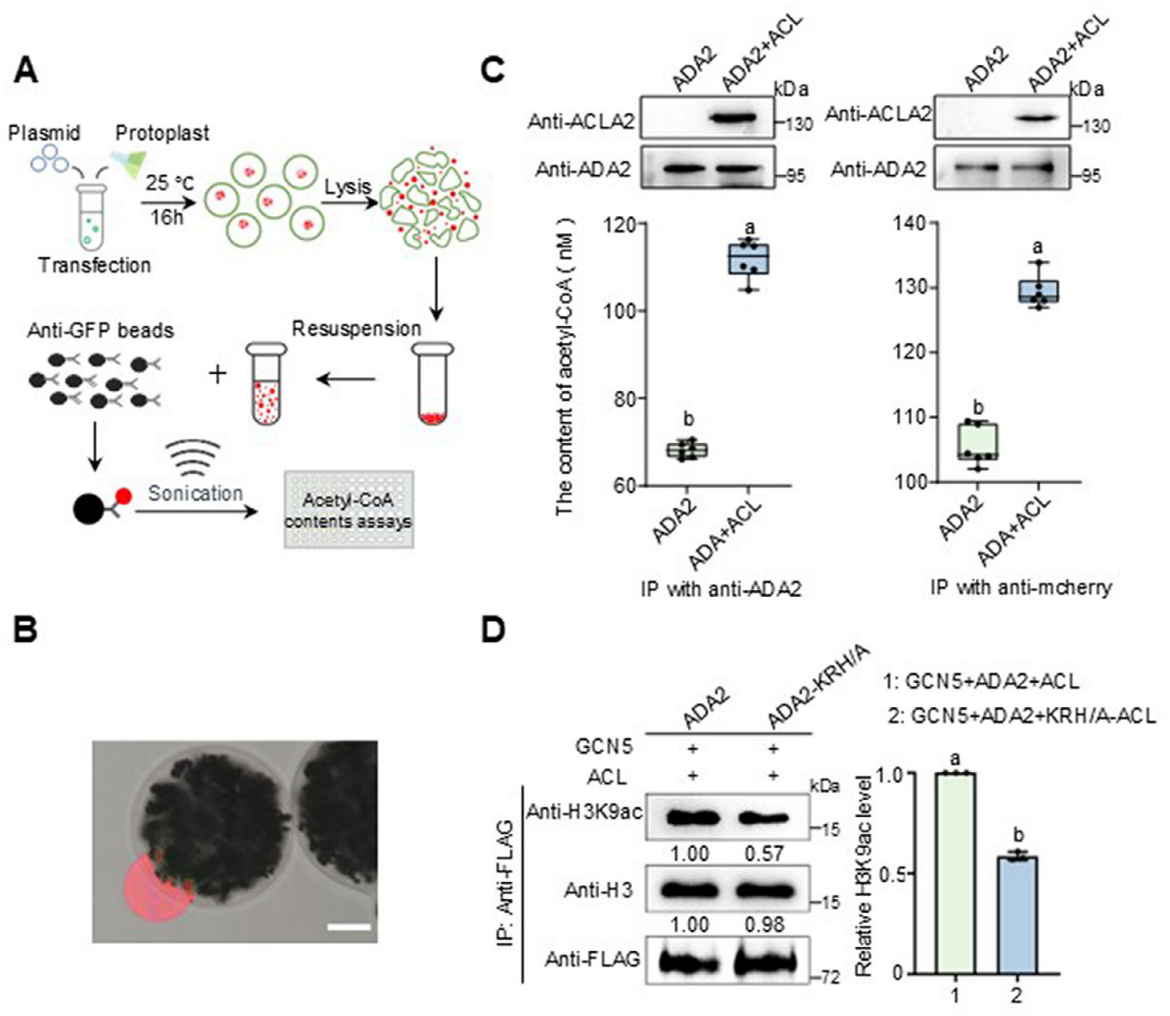
ACL enriches acetyl‐CoA in GAA complex condensates. A) Flow chart showing acetyl‐CoA concentration analysis for the ADA2 condensate in this study (see methods). B) Image shows the immunoprecipitated condensate of ADA2‐mCherry by antibody‐coated magnetic beads (black sphere). Bar = 20 µm. C) Top panel, for benchmarking, immunoblots were applied for detecting ADA2‐mCherry and ACLA2 concentrations for the immunoprecipitated products in the two indicated groups (ADA2‐mCherry alone versus ADA2‐mCherry co‐expression with ACLA2). The results show comparable ADA2 concentrations within the two immunoprecipitation groups. Bottom panel, boxplots showing the acetyl‐CoA levels detected in the ADA2 phases. The 25th and 75th percentiles (box), median, and highest and lowest values are shown. The proteins were expressed in the rice protoplast system. Two‐week‐old acla2 mutant seedlings were used for the protoplast isolation. ACL, the fusion protein construct of ACLA2‐ACLB‐GFP (see methods). D) Immunoblots show K9 acetylation modification levels of the histone co‐immunoprecipitated by either the GCN5‐ADA2‐ACL or GCN5‐ADA2‐KRH/A‐ACL complex. Histone H3 was detected as a control. Immunoblotting band intensities were quantified using ImageJ, and the relative band signals to the control (the first lane) (set at 1) are indicated below each band. The statistical analysis (right panel) shows immunoblotting data from three independent replicates, with error bars indicating the mean ± SD. The significance of differences was analyzed using Student's t‐test. Significant differences (*p* < 0.05) between the groups are highlighted by different letters.

ATP‐Citrate lyase produces acetyl‐CoA by lysing citrate in cells,^[^ ^22^, ^32^, ^33^ ^]^ we hypothesized that ACL within the GAA condensates plays a role in enriching acetyl‐CoA in the microenvironment of the droplets, potentially enhancing the acetylation of target genes. To validate this hypothesis, ADA2‐mCherry and ACLA2‐ACLB‐GFP were co‐expressed in *acla2* protoplasts with a control expressing only ADA2‐mCherry (see methods). After overnight culture, cells were lysed, and protein condensates were isolated and captured via anti‐ADA2 or anti‐mCherry immunoprecipitation (IP) (**Figure** **5**A,B) (see methods). Significant increases in acetyl‐CoA levels were detected in the ADA2‐ACL co‐expressing droplets compared to those containing only ADA2 (Figure 5C), suggesting that ACL indeed augments acetyl‐CoA concentration in GAA condensates.

Subsequently, we co‐expressed GCN5, ADA2‐FLAG (or ADA2‐KRH/A‐FLAG), and ACLA2‐ACLB within the tobacco system (see method). The histones bound by the GAA complex were co‐immunoprecipitated using anti‐FLAG beads and tested by Western blots. As shown in Figure 5D, the acetylation at K9 of histone H3 pulled down by ADA2‐KRH/A is significantly decreased compared to that pulled down by the complex with ADA2. These data suggest that the disruption of phase separation in the GAA complex directly impacts HAT activity, supporting the hypothesis that the condensate microenvironment benefits the enrichment of acetyl‐CoA and histone acetylation via ACL.

### 2.6 The GAA Complex Affects the Transcription of Functional Genes in Rice Root Tip and Inflorescence

**Figure 6:**
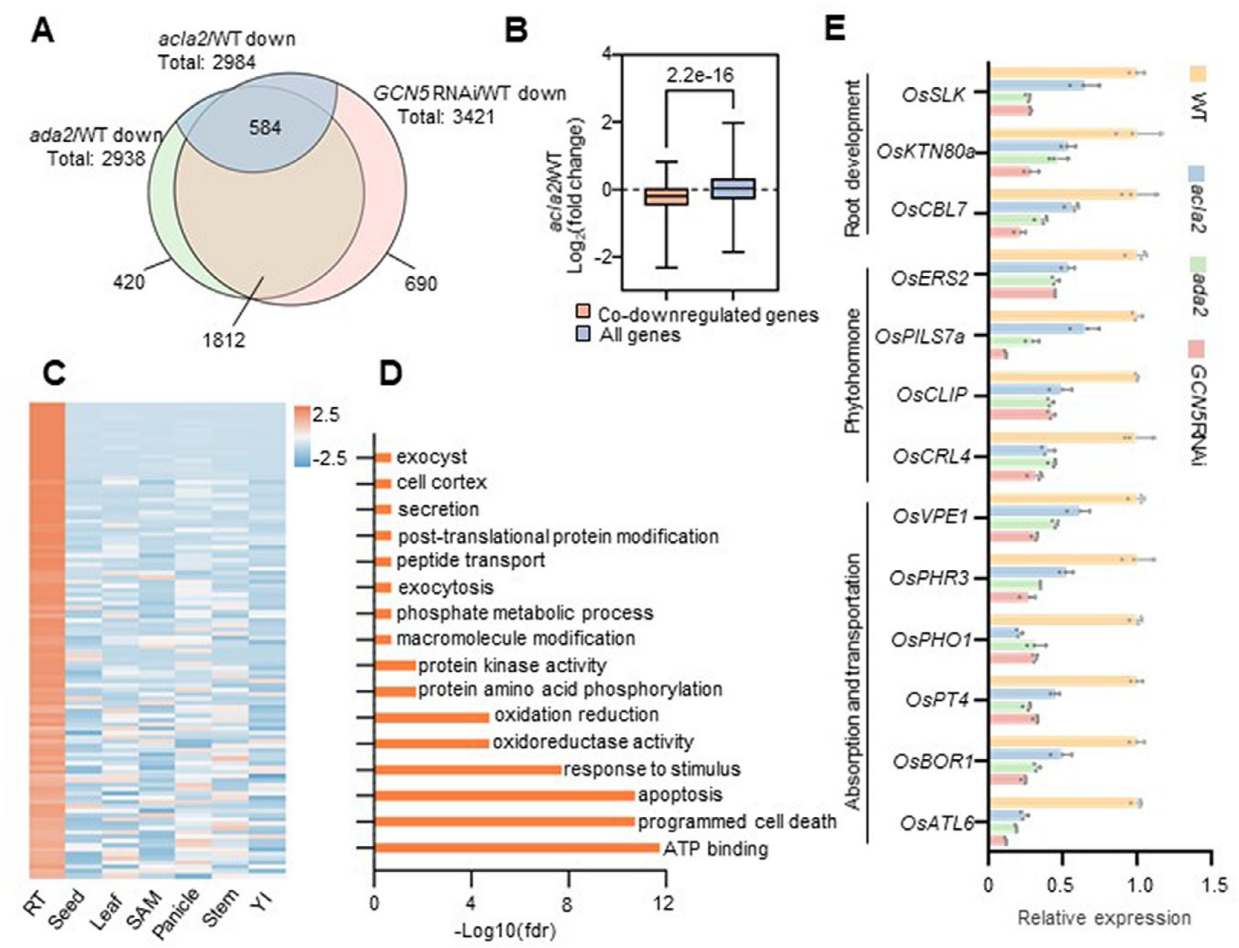
The GAA complex impacts gene transcription in root tips. A) Overlap of down‐regulated genes in *GCN5* RNAi, *ada2*, and *acla2* compared to wild type. Fold changes are larger than 2 *(GCN5* RNAi and *ada2)* and 1.5 *(acla2)*, respectively, with *q*‐value<0.05. B) Boxplots showing that co‐downregulated genes of *GCN5* RNAi and *ada2* have a down‐regulated trend in *acla2 *relative to genomic genes. *n* = 584 (Co‐downregulated genes), 19 131 (All genes). The 1%–99% percentile (box), median, and highest and lowest values are shown. Significant difference (Student's t‐test) between the two groups is indicated. C) Heat maps showing transcription profiles of genes specifically expressed at root tips (RT). SAM, Shoot apical meristem, YI, Young inflorescence. D) Gene ontology assay for the co‐downstream genes (*n* = 584) of GCN5, ADA2, and ACLA2. E) Histograms showing gene transcription levels in the indicated backgrounds. The values indicate the relative transcription levels compared to the wild type, with the wild type set as 1. Error bars represent the means ± SD from three independent biological replicates.

Given the notable deficiencies in root development observed in knock‐out or knock‐down lines of the three GAA components (Figure 2D–H), we profiled the root tip transcriptomes of *GCN5* RNAi, *ada2, acla2*, and wild‐type plants. Three biological replicates were conducted, and more than 65 million average clean reads were obtained for each sample (Table S1, Supporting Information). In sum, 3421 and 2938 (fold change>2, *q‐*value<0.05) genes were identified to be downregulated in *GCN5* RNAi and *ada2* compared to wild type, respectively (**Figure** **6**A), which were considered to be the direct downstream genes for the two proteins. Among them, 2396 (1812+584) genes (>70% downregulated genes) were commonly downregulated in both *GCN5* RNAi and *ada2*. Additionally, we observed a down‐regulation trend in the *acla2* mutant for the 2396 co‐downstream genes identified in *GCN5* RNAi and *ada2* (Figure 6B), implying the requirement or participation of ACLA2 in GCN5‐ADA2 activity for the transcription regulation process. Precisely, 2984 downregulated genes (fold change>1.5, *q‐*value<0.05) were identified in *acla2* relative to wild type, among which 584 genes were commonly downregulated in all three mutant/RNAi backgrounds (Figure 6A), which were considered as the downstream genes of the GAA complex for the following analysis. Regarding the upregulated genes, 468 co‐regulated genes were identified, which were found to be enriched in several gene ontology terms, such as ribosome metabolism, transcription regulation, and fatty acid metabolic process (Figure S8, Supporting Information).

Among the 584 genes, we observed an enrichment (18.8%, *P‐*value = 2e‐45, *Fisher's exact test*) of root tip specifically expressed genes compared to the whole genomic background (3.5%) (Figure 6C). Gene ontology analysis showed that genes involved in secretion, exocytosis, apoptosis, stimulus response, kinase activity, and catalytic activity were enriched in the co‐downstream genes (Figure 6D). Several genes, like *SLK, OsKTN80a, OsCBL7*,^[^ ^37^, ^38^, ^39^ ^]^ which have been reported to function in rice root development, were found to be regulated by the GAA complex. In addition, phytohormone‐related genes, such as *OsERS2* (ethylene receptor), *OsPILS7a* (auxin efflux carrier)*, OsCLIP* (cyto‐auxin transporter)*, OsCRL4* (cytokinin receptor),^[^ ^40^, ^41^, ^42^, ^43^ ^]^ and inorganic/organic elements absorption and transportation‐related genes, such as *OsVPE1* (vacuolar phosphate efflux transporter)*, OsPHR3* (phosphate response ortholog)*, OsPHO1* (phosphate transporter)*, OsPT4* (phosphate transporter)*, OsBOR1* (boron efflux transporter), and *OsATL6* (amino acid transporter),^[^ ^37^, ^44^, ^45^, ^46^, ^47^, ^48^ ^]^ were all downregulated in *GCN5* RNAi, *ada2*, and *acla2* (Figure 6E). These findings suggested that the three components of the GAA complex have a function in maintaining the transcription of functional genes in rice root tips.

By comparing the misregulated genes in *hag704* roots, another histone acetyltransferase that also interacts with ACLA2 and impacts root development,^[^ ^21^ ^]^ we observed relatively little overlap between the downregulated genes of the two HAT mutants (Figure S9A, Supporting Information). Similarly, the key genes regulated by the GAA complex are rarely downregulated in *hag704* compared to wild type (Figure 6E; Figure S9B, Supporting Information). In addition, unlike GAA, HAG704‐ACL is mainly involved in H4K5 acetylation required for the cell cycle.^[^ ^21^ ^]^ The distinct patterns of gene regulation suggest that these two complexes govern different genomic regions.

Moreover, we inspected the transcriptomes of inflorescence meristems from the three indicated mutant/RNAi materials as well as from the wild type. In total, 214 (120+94) genes (fold change>2, q‐value<0.05) were identified as being downregulated in *ada2* and *GCN5* RNAi compared to the wild type, among which 56% (120/214) (fold change>1.5, *q‐*value<0.05) were co‐regulated by *acla2* (Figure S10A, Supporting Information). Within these 120 co‐regulated genes, 14 genes were identified as being expressed specifically in the inflorescence meristem (Figure S10B, Supporting Information). Gene ontology analysis revealed that genes involved in transcriptional regulation and metabolic processes (e.g., nitrogen compound metabolism, RNA biosynthetic process) were enriched (Figure S10C, Supporting Information). Additionally, genes such as *OsBELL4B*, *OsEU1*, *OsGATA7*, and *OsDREB1C*, which have been reported to be involved in rice panicle development,^[^ ^49^, ^50^, ^51^, ^52^ ^]^ were found to be misregulated in all three mutant/RNAi backgrounds compared to the wild type (Figure S10D, Supporting Information). These data suggest that the GAA complex functions in maintaining key genes for inflorescence development.

### 2.7 The GAA complex Regulates Genomic Acetylation at H3K9, Impacting Gene Transcription

**Figure 7:**
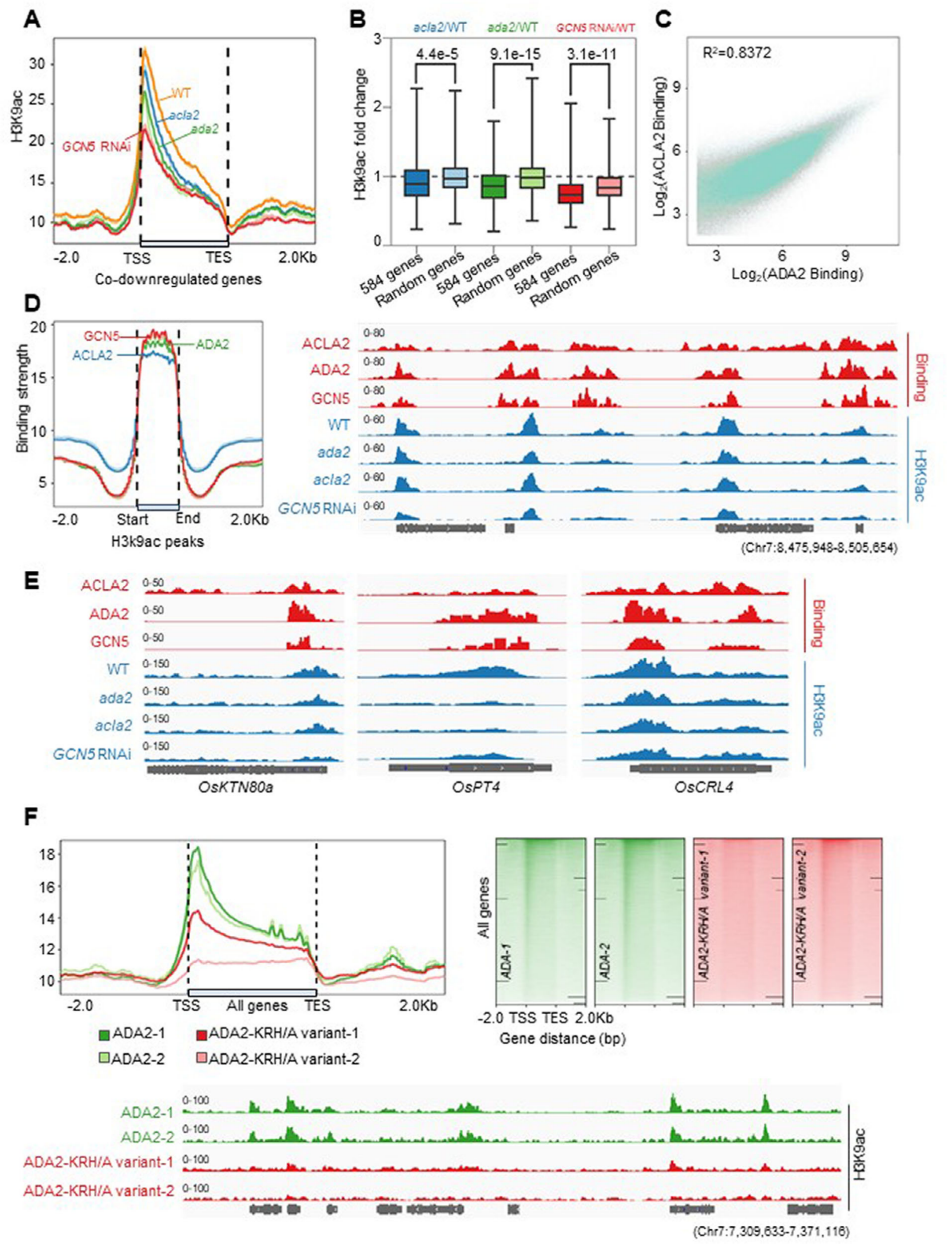
The GAA complex affects genomic H3K9 acetylation. A) H3K9 acetylation metaplots of co‐regulated genes of GCN5, ADA2, and ACLA2. Two biological replicates are shown. B) Box plots depicting the ratio of H3K9ac modification fold change in the mutant or RNAi backgrounds compared to wild‐type for the 584 co‐downregulated genes. 584 randomly selected genes were used in this study. The 25th and 75th percentiles (box), median, and highest and lowest values are shown. *P*‐values (Student's t‐test) were indicated. C) Scatterplots showing the correlation of the genomic binding strength between ACLA2 and ADA2. The squared Pearson correlation coefficient (R^2^) between the two variables is shown. D) Metaplots showing the bindings of ADA2, ACLA2, and GCN5 are enriched at H3K9ac peaks. IGV screenshot exhibits a positive correlation between ADA2, ACLA2, GCN5 binding, and H3K9ac modification. E) IGV screenshots show the binding signals for the three indicated GAA components and the H3K9ac modification status in the specified backgrounds. The three genes are reported to be involved in root development. F) Metaplots illustrate the genomic average H3K9 acetylation levels in the *ada2* protoplasts transiently expressing either ADA2‐GFP or ADA2‐KRH/A variant‐GFP. Heat maps show the H3K9 acetylation levels along all genomic genes. IGV screenshot exhibits the H3K9ac status for a typical genome region.

Our previous work has shown that rice GCN5 could acetylate multiple sites on the histone H3 tail in vitro, such as K9, K14, and K27.^[^ ^11^ ^]^ This led us to investigate whether the GAA complex could directly bind to the targets and regulate their chromatin status and corresponding transcription. The genomic H3K9 acetylation (H3K9ac) of root tips was profiled via the CUT&Tag approach in *GCN5* RNAi, *ada2*, *acla2*, and wild‐type plants (Table S2, Supporting Information). By investigating the 584 co‐downstream genes, we found that H3K9ac was significantly attenuated in all three mutant/RNAi backgrounds compared to the wild type (**Figure** **7**A). In addition, statistics showed that the attenuation fold change of the 584 commonly regulated genes was noticeably larger than that of randomly selected H3K9ac‐marked genes, suggesting that the GAA complex has a much more significant impact on the 584 common target genes in rice root tips relative to the genomic background (Figure 7B).

Subsequently, we investigated the genomic binding of all three GAA components via CUT&Tag with tag‐fusion transgenic lines. As shown in Figure 7C; Figure S11A (Supporting Information), the genomic distributions of GCN5, ADA2, and ACLA2 resembled each other (see methods), with R‐squared values larger than 0.75, in accord with the finding that GCN5, ADA2, and ACLA2 work as a complex. Moreover, we found that the three protein‐binding signals were enriched at the H3K9ac peaks, underscoring their regulatory role in H3K9 acetylation (Figure 7D). Among them, functional key genes involved in rice root development, such as *OsKTN80a*, *OsPT4*, and *OsCRL4*,^[^ ^38^, ^43^, ^47^ ^]^ which were previously found to be downstream genes of the GAA complex (Figure 6E), were found to be directly bound and acetylation‐regulated by the GAA complex (Figure 7E; Figure S11B, Supporting Information).

Investigating the sequence nature of the GAA binding regions, we identified several enriched motifs, including “TTAATGG”, “CGCCGCC” (Figure S11C, Supporting Information). Among these, the “TTAATGG” motif is known as the binding site of WOX11,^[^ ^53^ ^]^ and the latter sequence resembles the motifs of the transcription factor, SHN3 (AP2 protein). These data imply their potential roles in recruiting the GAA complex to target genomic regions.

To validate the impact of phase separation of the GAA complex on genomic H3K9 acetylation, we transiently expressed ADA2‐GFP and the ADA2‐KRH/A‐GFP variant in protoplasts derived from the *ada2* mutant. The latter variant was unable to mediate condensation of the GAA complex without significantly affecting the integrity of the HAT module (Figure S6A, Supporting Information). Genomic profiling revealed that H3K9ac in cells expressing the ADA2‐KRH/A‐GFP variant was remarkably weaker than that in cells expressing ADA2‐GFP (Figure 7F), indicating that the abolition of GAA complex phase separation largely affects histone acetylation, highlighting the necessity of the nuclear GAA complex fusion in regulating chromatin status.

### 2.8 The Phase Separation of ADA2 Conserved Among Species

Analysis of ADA2 homologs from other species revealed that they exhibit varying lengths of intrinsically disordered regions with an enrichment of charged amino acids compared to their proteome background, as seen in rice (Figure S12A,B; Figure S3A, Supporting Information). Testing representative ADA2 homologs from other species in the tobacco system revealed a noticeable condensation ability of the protein (Figure S12C, Supporting Information), implying the conservation of condensation behavior among plant ADA2 homologs.

## 3 Discussion

The SAGA complex is a highly conserved histone acetyltransferase complex found in eukaryotes, ranging from unicellular organisms like yeast to higher animals and plants.^[^ ^1^, ^2^, ^3^, ^4^ ^]^ This complex plays a critical role in regulating chromatin acetylation status, which in turn impacts gene transcription. Based on the classic understanding of the SAGA complex,^[^ ^54^, ^55^, ^56^, ^57^ ^]^ our work unveiled a novel mode of action of the complex in modulating chromatin modifications and gene regulation. Specifically, we identified that the core component ADA2 harbors an intrinsically disordered region (IDR) enriched with charged amino acid residues, which likely directs the entire complex to undergo condensation within the nucleus.

Growing evidence indicates that the formation of specific genomic features in the nucleus is at least partially driven by the condensation or phase separation of diverse proteins. For instance, the histone variant H2A.W enhances chromatin condensation by promoting chromatin fiber interaction via its conserved C‐terminal motif.^[^ ^58^ ^]^ In sperm cells, H2B.8 was identified to be deposited in the lowly expressed genomic regions, driving chromatin phase separation to condense the sperm nucleus.^[^ ^59^ ^]^ In addition to histone variants, BP1, a homeodomain domain‐containing protein, recognizes trimethylation of H3K27, was found to form phase‐separated liquid condensates to maintain transcriptional repression in *Fusarium graminearum*.^[^ ^60^ ^]^ Additionally, phase‐separated CBXs/PHCs in mammalian cells drive the formation of Polycomb‐repressive complex 1 (PRC1) condensate, thereby enhancing chromatin compaction and/or PRC1 ubiquitination activity.^[^ ^61^, ^62^ ^]^ In our study, alongside the suppressive markers, we provide another example illustrating how active genomic features are formed and maintained through the phase separation of epigenetic regulators.

Despite observing liquid‐like condensates for ADA2 and the GAA complex in vivo (Figure 3F; Figure S5, Supporting Information), we have not yet developed an effective method to achieve mobility for ADA2 droplet in vitro (Figure S2C, Supporting Information). Although we have tried various buffers, pH adjustments, and co‐expression with other GAA components (data not shown), these attempts have not been successful. This suggests that the microenvironment responsible for ADA2 dynamics is still unknown.

Moreover, despite observing phase separation of ADA2 in root tips (Figure 3D; Figure S2A, Supporting Information), we failed to observe obvious protein aggregation behavior within the nuclei of the elongation zone (Figure S2D, Supporting Information). This indicates that the phase separation of the GAA complex is limited to the meristem zone in rice, potentially benefiting the transcription of key genes within this zone rather than in other regions. The mechanisms underlying the dynamics of ADA2 phase separation behavior in vivo remain elusive and require further investigation.

Previous reports indicate that ACLA2 interacts with HAG704, another histone acetyltransferase in rice.^[^ ^21^ ^]^ Combining these findings with our current work suggests that ACL is broadly engaged in histone acetylation reactions. However, given the condensate nature of the GAA complex in the nucleus (Figure 4), the cooperation between GCN5‐ADA2 and ACL appears to provide a more efficient mechanism for creating a microenvironment enriched with acetyl‐CoA to support histone acetylation reactions (Figure 5).

It has been reported that the fluctuation of acetyl‐CoA within cells can determine the lysine acetylation of ADA2, which consequently leads to protein ubiquitination and degradation.^[^ ^12^ ^]^ Given the stimulatory effect of ADA2 acetylation on phase separation (Figure S4B, Supporting Information), we hypothesize that the strong aggregation effect directed by protein acetylation may play a role in ADA2 degradation by facilitating protein gathering. How acetyl‐CoA fluctuations modulate genomic histone acetylation via the GAA aggregation dynamics requires further evidence in future investigations.

In summary, this study has unveiled a novel mechanism by which the SAGA complex maintains H3K9 acetylation to modulate gene expression involved in the development of rice roots and panicles. Within this mechanism, the core SAGA components, GCN5‐ADA2, recruit a citrate lyase unit (ACL) and condense in the nucleus via ADA2 phase separation, forming a microenvironment that facilitates histone acetylation and the transcription of functional genes essential for rice meristem activities (Figure S13, Supporting Information).

## 4 Experimental Section

### Plant Materials and Growth Conditions

In this study, the rice variety Zhonghua11 (*Oryza sativa ssp. Japonica*) was used to produce the transgenic plants. For in vitro cultures, seeds were surface‐sterilized and germinated in media containing 0.3% phytagel supplemented with 2% (w/v) sucrose at 28°C (in light) and 24°C (in dark) with a 14 h light/10 h dark cycle. Tobacco (*Nicotiana benthamiana*) plants used for protein transient expression were grown in soil for 6 weeks at 20 ± 2°C, 8 h photoperiod, and 100 µmol quanta/ (m2 s) illumination. All indicated rice plants were grown in the Wuhan area during the summer rice growing season.

### Rice Transgenic Plants and Vector Construction

The *acla2*, *ada2*, *GCN5* RNAi, and *Ubipro::GCN5‐2×FlAG‐2×HA* transgenic plants used in this study were characterized in a previous study,^[^ ^11^, ^12^, ^21^ ^]^ respectively. For the aggregation observations of ADA2, the *Ubipro::ADA2‐GFP*, *ADA2pro::ADA2‐GFP*, and *ADA2pro::ADA2‐ΔIDR2‐Venus* (deletion of IDR2) transgenic plants were generated by fusing GFP or Venus tag to the C‐terminus of the genome sequences, driven by either *Ubiquitin* (*Ubi*) or *ADA2* (2.6 kb) promoter. For detecting the bindings of ACLA2 and GCN5 in the genome, *Ubipro::ACLA2‐FLAG* and *GCN5pro::mCherry‐GCN5* transgenic plants were generated. For detecting the binding of ADA2, the *ADA2pro::ADA2‐GFP* mentioned above was utilized. Transgenic lines of rice were generated via the reported method.^[^ ^63^, ^64^ ^]^

### For Yeast Two‐Hybrid Analysis

Rice *ACLA2* cDNA was cloned into the pGADT7 (Clontech), with *ADA2*, *ACLB* cDNAs were cloned into the pGBKT7 (Clontech) vector. Yeast cells containing the pGBKT7 and pGADT7 plasmids were grown on stringent selection medium (synthetic dropout medium lacking Leu, Trp, His, and adenine (‐LTHA) or non‐selective medium lacking Trp and Leu (‐LT) as a control.

### For In Vitro Pull‐Down Assay

The His‐tagged ADA2 and GST‐tagged ACLA2 were expressed in *Escherichia coli*. GST or ACLA2‐GST coupled with GST beads (GE Healthcare, 17‐5132‐01) was used to pull down ADA2‐6×His. His (1:4000, Abcam, ab9108) and GST antibody (1:4000, Abcam, ab19256) were applied for western blot detection.

### For the split‐Luciferase Complementation Assays

The cDNA of *ACLA2*, *ACLB*, and *GCN5* were cloned in‐frame upstream of the sequence encoding the N‐terminal half of firefly Luciferase (NLuc), while that of ACLA2, ADA2, or ADA2 variants (ADA2‐KRH/A, ADA2‐DE/A) were cloned in‐frame downstream of the sequence encoding the C‐terminal half of firefly Luciferase (CLuc). Subsequently, the constructed vectors were co‐infiltrated into 6‐week‐old tobacco (*Nicotiana benthamiana*) leaves in different combinations. After 48 h of transfection, tobacco leaves were infiltrated with 1 mM luciferin (Gold Biotechnology, 115144‐35‐9). Luciferase bioluminescence images were taken with the Chemi‐Image System (Tanon 5200Multi, China).

### For Co‐Immunoprecipitation Assay in Rice Protoplasts

The *35Spro::ADA2‐FLAG* construct was transfected into rice protoplasts with *35Spro::ACLA2‐GFP* or GFP alone. After 12 h of incubation, the protoplasts were harvested and lysed in RIPA buffer (10 mM Tris‐HCl, pH 7.5, 150 mM NaCl, 0.5 mM EDTA, 0.1% [w/v] SDS, 1% [v/v] Triton x‐100, 1% [w/v] sodium deoxycholate, 2.5 mM MgCl_2,_ 1 mM PMSF) for 30 min. Then, anti‐GFP M2 magnetic beads (AlpalifeBio, KTSM1334) were incubated with the protoplast supernatant overnight at 4 °C, and the proteins were then precipitated and analyzed by immunoblot with anti‐GFP (1:1000, Abcam, ab290) and anti‐FLAG (1:1000, Sigma, F3165) antibodies.

### For Co‐Immunoprecipitation Assay in GCN5 Overexpression Plants

The 14‐day‐old Seedlings were ground into power by liquid nitrogen and lysed in RIPA buffer (10 mM Tris‐HCl, pH 7.5, 150 mM NaCl, 0.5 mM EDTA, 1% [w/v] SDS, 1% [v/v] Triton x‐100, 1% [w/v] sodium deoxycholate, 2.5 mM MgCl_2_, 1 mM PMSF) for 30 min, followed by centrifugation at 12000 g for 10 min to remove cellular debris at 4 °C. The supernatant was transferred to a new tube and incubated with anti‐FLAG M2 magnetic beads (Sigma, M8823) overnight at 4 °C. After three washes with washing buffer (10 mM Tris‐HCl pH 7.5, 150 mM NaCl, 0.5 mM EDTA), the co‐immunoprecipitated proteins were separated by SDS‐PAGE and detected with anti‐HA (1:5000, ABclonal, AE036), anti‐ACLA2^[^ ^21^ ^]^ (1:1000) and anti‐ADA2^[^ ^11^ ^]^ (1:1000) antibodies.

### Rice Root Tip Clearing

Rice root tips (1–1.5 cm) were placed in the center of the slide and treated for 12 h with a transparent agent (Chloral hydrate 8 g, ddH_2_O 3 mL, glycerol 1 mL), and then observed with a Nikon Ni‐E light microscope with a CCD camera. The length of the meristematic zone was determined by counting cells starting from the quiescent center until the end of the last cell in the meristematic region (the length of the meristematic cells is approximately half that of the elongation region). The total length was measured from stitched images of consecutive microscopic fields and converted to actual length using the scale bar.

### Edu Staining

EdU staining was conducted using an EdU kit (Ribobio, C10310) as described previously.^[^ ^11^ ^]^ Roots of three‐day‐old (post‐germination) seedlings were submerged in a 50 µM EdU solution for 3 h. After 30 min of fixation in 4% paraformaldehyde, a longitudinal vibration slice was collected and treated with Apollo staining solution. The florescence was detected with a laser confocal microscope (Olympus, FV1200).

### Prediction for Disordered Region

Disordered region prediction was performed using the online Predictor of Natural Disordered Regions (PONDR) database (http://pondr.com/). Regions with an average strength (PONDR score) ≥ 0.5 were considered to be disordered.

### Imaging of ADA2 and ADA2 Variants In Vivo

To investigate the gathering manner of ADA2 in rice protoplasts, the constructs, *35S::ADA2‐GFP* or *35S::ADA2‐ΔIDR‐GFP*, were co‐transformed within the rice protoplast system with *35S:SRT2‐mCherry* (a nucleus maker).^[^ ^65^, ^66^ ^]^ After 12 h of culture, cells were collected and imaged with a Zeiss LSM980 confocal microscope. For tobacco protein distribution imaging, the coding sequences of ADA2 and its variants (or homologs from other species) were cloned into pCAMBIA1300‐35S‐GFP vector. 6‐week‐old tobacco leaves were used for Agrobacterium injection. After 48 h of transfection, the florescence was detected with a Zeiss LSM980 confocal microscope. For imaging of transgenic lines, root tips from 2‐week‐old seedlings of *Ubipro::ADA2‐GFP*, *ADA2pro::ADA2‐GFP*, and *ADA2‐ΔIDR2‐Venus* transgenic lines were collected for imaging.

### For Condensate Occupation Area Assay

The droplet area was measured by ImageJ (v1.8.0_172), 45samples are used for statistical analysis.

### In Vitro Phase Separation Assay

For the in vitro phase separation assay, rice ADA2‐GFP and GFP were cloned into the pGEX‐6p vector. Proteins (GST‐ADA2‐GFP and GST‐GFP) were induced and expressed in *E.coli* BL21 (DE3) cells at 16°C by applying 0.2 mM IPTG. After overnight incubation, cells were collected, resuspended in pull‐down buffer (20 mM Tris‐HCl (pH 8.0), 200 mM NaCl, 1 mM EDTA, 0.5% NP40, 1 mM PMSF), and sonicated. Subsequently, the supernatant was collected by centrifugation at 12 000 g, 4°C for 5 min. After incubation with washed GST beads for 12 h, the precipitated protein was eluted in GST elution buffer (50 mM Tris‐HCl (pH 8.0), 150 mM NaCl, 10 mM glutathione reduced). For the in vitro phase separation assay, purified proteins of GST‐ADA2‐GFP or GST‐GFP were mixed with PEG8000 to achieve a final concentration of 10% (w/v) (Sigma, P5413) as reported^[^ ^16^ ^]^ and imaged by utilizing a Zeiss LSM980 microscope.

### Fluorescence Recovery After Photobleaching (FRAP)

FRAP of ADA2‐GFP and GAA (GCN5‐ADA2‐ACLA2) condensates in the nucleus of tobacco epidermal cells was performed on a Zeiss LSM980 confocal microscope. The region of ADA2‐GFP and GAA condensates was bleached using a 488‐nm laser pulse (10 iterations, 100% intensity) or 405/488/594‐nm laser pulse (15 iterations, 100% intensity), respectively. Fluorescence recovery was recorded every 1 s for 40 or 140 s after bleaching. Images were acquired using ZEN software. The fluorescence recovery values were normalized by the first post‐bleach time point and divided by the maximum point set maximum intensity as 1.

### For In Vitro FRAP

Samples mixed with PEG8000 (10% m/v) were mounted on slides, and the FRAP was conducted by a ZEN microscope equipped with 60× oil immersion objectives.

### Concentration Measurement of Acetyl‐CoA in ADA2 Condensates

mCherry‐tagged ADA2 protein was co‐expressed with or without GFP‐tagged ACL protein (ACLA2‐ACLB‐GFP, cascade connection of the two ACL components mimics the full length of ACL in mammalian cells, which is thought to have complete enzymatic activity^[^ ^22^ ^]^) for 16 h in the protoplasts derived from *acla2* mutant. Subsequently, the protoplasts were collected and mixed with mild lysis buffer (NEBII buffer:0.25 M sucrose, 10 mM Tris‐HCl, pH 8.0, 1 mM MgCl_2_, 0.1% [v/v] Triton x‐100) at 4°C for 30 min, followed by centrifugation at 300 g, 4°C for 5 min to remove cell debris. The supernatant was transferred to a new 1.5 ml tube and centrifuged at maximal speed (16 873 g), 25°C for 15 min to enrich the ADA2 phase. The collections were resuspended in the column buffer (50 mM Tris‐HCl, pH 7.5, 100 mM NaCl, 1 mM EDTA, 5 mM DTT) as reported.^[^ ^67^ ^]^ Two independent methods are applied to immunoprecipitate the ADA2 droplets by either anti‐ADA2 coated protein‐A magnetic beads (Thermo Fisher Scientific, 10001D) or anti‐mCherry M2 magnetic beads (ABMagic, MA111‐25T). After two washes with the indicated buffer, the beads were harvested and resuspended in an equal volume 1×PBS (pH 7.2–pH 7.4). 30 µL beads for each reaction were taken out and mixed with some volume 1×SDS loading buffer (62.5 mM Tris‐HCl pH 6.8, 10% glycerol, 1.25% SDS, 0.1% [W/V] bromophenol blue, and 1.25% [V/V] β‐mercaptoethanol) and denatured at 95°C for 10 min for further immunoblot. The Western Blots using anti‐ADA2 (1:1000) and anti‐ACLA2 (1:1000) antibodies were used to indicate the concentrations of immunoprecipitated proteins. The rest magnetic beads were sonicated 10 times to release the immunoprecipitated droplets, and the supernatant was used for the acetyl‐CoA assay. The acetyl‐CoA was extracted as previously described^[^ ^35^ ^]^ and measured by utilizing an acetyl‐CoA assay kit (Ji Ning Biotech, JN709212). The antibodies for ADA2 and ACLA2 were from the reported works.^[^ ^11^, ^21^ ^]^

The constructs of 35S::GCN5::NOS, 35S::ADA2‐flag::NOS (or 35S::ADA2‐KRH/A‐flag::NOS), and 35S::ACLA2‐ACLB::NOS were cloned into one vector, and transformed into tobacco cells via agrobacteria to ensure the co‐expression of the three specified proteins. After two days of culture, the histones immunoprecipitated by anti‐flag beads were extracted from the infected leaves, and were tested using immunoblots.

### In Situ Hybridization Assay

The hybridization and immunological detection were conducted as described by previously reported.^[^ ^53^ ^]^ Linearized DNA templates containing ACLA2‐specific sequences were transcribed in vitro using SP6 or T7 promoters to produce digoxigenin‐labeled sense and antisense RNA probes (Roche, 1 175 025). Additionally, ADA2 and GCN5 probes were designed as previously described^[^ ^11^ ^]^ and generated as above. RNA in situ hybridization was performed by utilizing the inflorescence meristems collected from the wild type. The process of RNA hybridization strictly adheres to the method described in the reported paper.^[^ ^53^ ^]^

### Paraffin Section Experiment

The rice inflorescence meristem was collected and fixed in FAA (4% formaldehyde, 10% acetic acid, and 50% ethanol) before being dehydrated in a series of graded ethanol concentrations. Subsequently, the tissue was subjected to gradient chloroform‐alcohol mixtures, followed by gradient chloroform‐paraffin infiltration. The tissue was infiltrated with molten paraffin twice and then embedded in wax. The embedded samples were sliced into 8 µm‐thick sections and counterstained with aniline blue solution (Aladdin, A113197). Images were captured using a Nikon Ni‐E light microscope.

### ADA2 Phylogeny Analysis

Homologs of rice ADA2 in other species were identified by the blast function in the National Center for Biotechnology Information (NCBI). The proteomes of different species were downloaded from the Universal Protein Database (UniProt). The protein domains were predicted by the Simple Modular Architecture Research Tool (SMART). The intrinsically disordered region of ADA2 was predicted by the Predictor of Natural Disordered Regions (PONDR) database (http://pondr.com/). The ADA2 proteins from rice and other species were aligned by ClustalX software. The MEGA (v5.2.2) program was used to conduct the phylogenetic analysis. The Neighbor‐Joining method was used to estimate evolutionary distances.

### RNA‐Seq Process and Data Analysis

Total RNA was isolated from either 1 cm rice root tips of 7‐day‐old post‐germination seedlings and 0.1–0.2 cm inflorescence meristems as reported^[^ ^68^ ^]^ using the RNeasy Plus Micro Kit (Qiagen, 74 034). First‐strand cDNA was synthesized from 100 ng of total RNA using a reverse transcription kit (Vazyme, N712). Sequencing libraries were then prepared with the Vazyme TD502 kit and sequenced on an Illumina HiSeq 3000 system. Raw RNA‐seq reads were quality‐filtered using FastP (v 0.23.4) to remove adapter sequences and low‐quality reads. Clean reads were aligned to the rice reference genome (MSU v7.0) using HISAT2 (v2.2.0). Differential gene expression analysis was performed with Cufflinks (v2.2.1). Genes with *p*‐value < 0.05 and fold change >2 (1.5 for *acla2*) in mutants relative to wild type were considered as differentially expressed genes. Normalized transcription profiles were generated by Cuffnorm (v2.2.1), including stem (SRR1777241, SRR1777242), root (SRR5296167, SRR5296168), leaf (SRR7516793, SRR7516794), seed (SRR8365239, SRR8365240, SRR8365241), young inflorescences (SRR16127055, SRR16127056), and shoot apical meristem (SRR23611909, SRR23611910, SRR23611911). The root tip specifically expressed genes were simply identified via the following criteria: RPKM (root tip)/RPKM (average level of other tissues)>5 & RPKM (root tip)/RPKM (maximum of other tissues)>2. The enrichment significance of root‐specific expressed genes relative to the genomic background was calculated by Fisher's exact test.

### CUT&Tag Process and Analysis

The CUT&Tag experiment was performed according to the instructions of the Hyperactive Universal CUT&Tag Assay Kit (Vazyme, TD903). Root tips from 7‐day‐old germinated rice seedlings were harvested to isolate nuclei. The nuclei were washed with wash buffer (Vazyme, TD903), followed by incubation with ConA Beads (Vazyme, TD903) at room temperature for 10 min. Consequently, the supernatant was replaced with 50 µL of primary antibody incubation buffer, and primary antibody was added at a 1:50 dilution. The mixture was incubated at 4°C overnight. The following day, the supernatant was discarded, beads were resuspended in dig‐wash buffer (Vazyme, TD903), and secondary antibody was added at a 1:100 dilution. After gentle mixing, the mixture was incubated at room temperature with rotation for 1 h. The beads were washed three times with 200 µL of dig‐wash buffer to remove unbound antibodies. The beads were resuspended with dig‐300 buffer (Vazyme, TD903), and pA/G‐Tnp Pro (Vazyme, TD903) was added at a 1:50 ratio. Following gentle mixing, the mixture was incubated at room temperature with gentle rotation for 1 h. The beads were washed three times with 200 µL of dig‐300 buffer and then incubated in TTBL buffer at 37°C for 1 h to perform the fragmentation reaction. The reaction was terminated by adding Buffer L/B (Vazyme, TD903) and Proteinase (Vazyme, TD903) to degrade proteins. Genomic DNA was purified using DNA Extract Beads (Vazyme, TD903). The purified DNA was amplified using i5 and i7 index primers (Vazyme, TD202) and 2×CAM (Vazyme, TD903). The PCR‐amplified library was purified using VAHTS DNA Clean Beads (Vazyme, N411‐01) and sequenced on the Illumina NovaSeq X Plus platform. Anti‐H3K9ac (ABclonal, A21107), anti‐GFP(ABclonal, AE078), anti‐mcherry (ABclonal, AE127), anti‐FDDK (Abmart, M20008) antibodies were used. Raw reads were filtered using FastP (v0.23.4) to eliminate adapter sequences and low‐quality reads. Clean reads were aligned to the rice reference genome (MSU v7.0) using Bowtie2 (v2.5.2). Then, duplicated reads were discarded using samtools (v1.9). SICER2 software (version 1.0.2) was used to call histone modification peaks with “‐w 200 ‐g 600 ‐f 150 ‐egf 0.74 ‐fdr 0.01” parameters. BigWig files generated by deepTools software with RPKM normalization were used for data visualization by IGV (version 2.3.88). Metaplots representing the average H3K9ac modification levels across different materials were generated using the computeMatrix module of deepTools (version 3.5.0). For H3K9ac fold change calculation, the H3K9ac strength within the 1 kb upstream and downstream regions of the transcription start site (TSS) for the 584 commonly regulated genes and 584 randomly selected genes was calculated using the multiBigwigSummary module of deepTools. For binding signal comparison, the binding strengths of ADA2, ACLA2, and GCN5 were calculated for each 500 bp genomic bin (a total of 748 950 bins in the rice genome) using the multiBigwigSummary module of deepTools.

### Motif Analysis

The overlapped genomic regions with binding signals of ADA2, GCN5, and ACLA2 were used. Motif analysis was performed using findMotifs.pl of the HOMER package (version 4.11).

### Statistics Analysis

The data are presented as mean ± standard deviation (SD). Statistical comparisons between two groups were performed using Student's t‐test (paired, two‐sided). One‐way ANOVA with Tukey's multiple comparison tests was applied for comparing three or more groups. *p* < 0.05 was considered statistically significant. Data analysis was conducted using Microsoft Excel (version 2304) and GraphPad Prism Software (Version 8.0.1). Detailed statistical information can be found in the figure legends. The RNA‐seq data generated in‐house consisted of two or three biological replicates for each genotype, and the CUT&Tag data comprised two biological replicates for each genotype. The biological replicates of all other experiments presented in this study are indicated in the respective figure legends.

## Conflict of Interest

The authors declare no conflict of interest.

## Author Contributions

Y.Y., T.L., and X.G. contributed equally to this work. All authors read and approved the final manuscript. Y.Y., T.L., and X.G. performed the experiments and analyzed the data. S.Z. and D.Z. supervised the project. H.A., X.L., R.Z., and J.G. participated in the project design. B.L. and S.L. participated in part of the experiments. S.Z. analyzed the data and wrote the paper.

## Supporting information



Supporting Information

Supporting Information

Supporting Information

Supporting Information

Supporting Information

Supporting Information

Supporting Information

## Data Availability

The gene information mentioned in this study is listed in Table S3 (Supporting Information). Gene information for these loci can be found on the Rice Genome Annotation Project website (http://rice.plantbiology.msu.edu/index.shtml). The gene sequences of CDS and protein, along with their corresponding annotations, are accessible via the “locus search” function on the website. Data supporting the findings of this work (RNA‐seq and CUT&Tag data) have been deposited into the Gene Expression Omnibus database under the accession number GSE300491. The processed data are listed in Tables S1 and S2 (Supporting Information). The primers used in this study are listed in Table S4 (Supporting Information). All the source data for the images and statistics in this work are provided (Source data of figures.pdf, Source data of statistics.xlsx).
