# Supplementary material for: ADA2 Forms Nuclear Condensates with GCN5 and ATP‐Citrate Lyase (ACL) to Modulate H3K9 Acetylation at Genes Functioning in Rice Meristems
Source: Adv Sci (Weinh). 2025 Nov 12;13(5):e13169. doi: 10.1002/advs.202513169 (PMC12849889; doi:10.1002/advs.202513169)
Supplement: Supplementary file 1 — Supporting Information [file ADVS-13-e13169-s007.pdf]

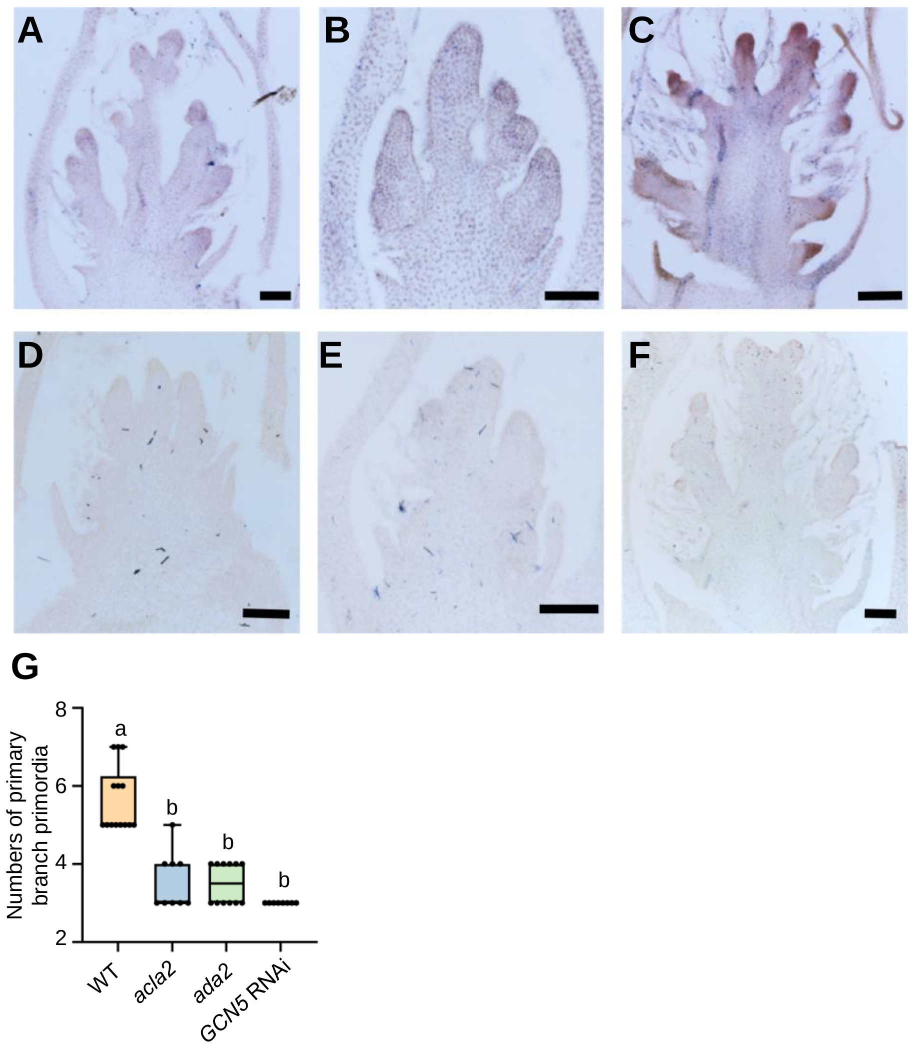


**Figure S1.** *In situ* hybridization of *ACLA2*, *ADA2*, *GCN5* transcripts in inflorescence meristem (IM). A-F) Images showing the hybridizations of inflorescence meristems with antisense probes for *ACLA2* (A), *ADA2* (B), *GCN5* (C) and sense probes for *ACLA2* (D), *ADA2* (E), *GCN5* (F). Bars = 100 μm. G) Boxplots showing the number statistics of primary branch primordia for the indicated backgrounds. n = 14 (WT), 9 (*acla2*), 12 (*ada2*) and 8 (*GCN5* RNAi). The boxplots show the 25th and 75th percentiles (box), median and highest and lowest values. Error bars represent the means ±SD from independent biological replicates. The different significances were calculated using one-way ANOVA with Tukey's multiple comparison tests. Different letters on top of the bars indicate a significant difference (p-value < 0.05), and the same letters on top of bars indicate no significant difference.

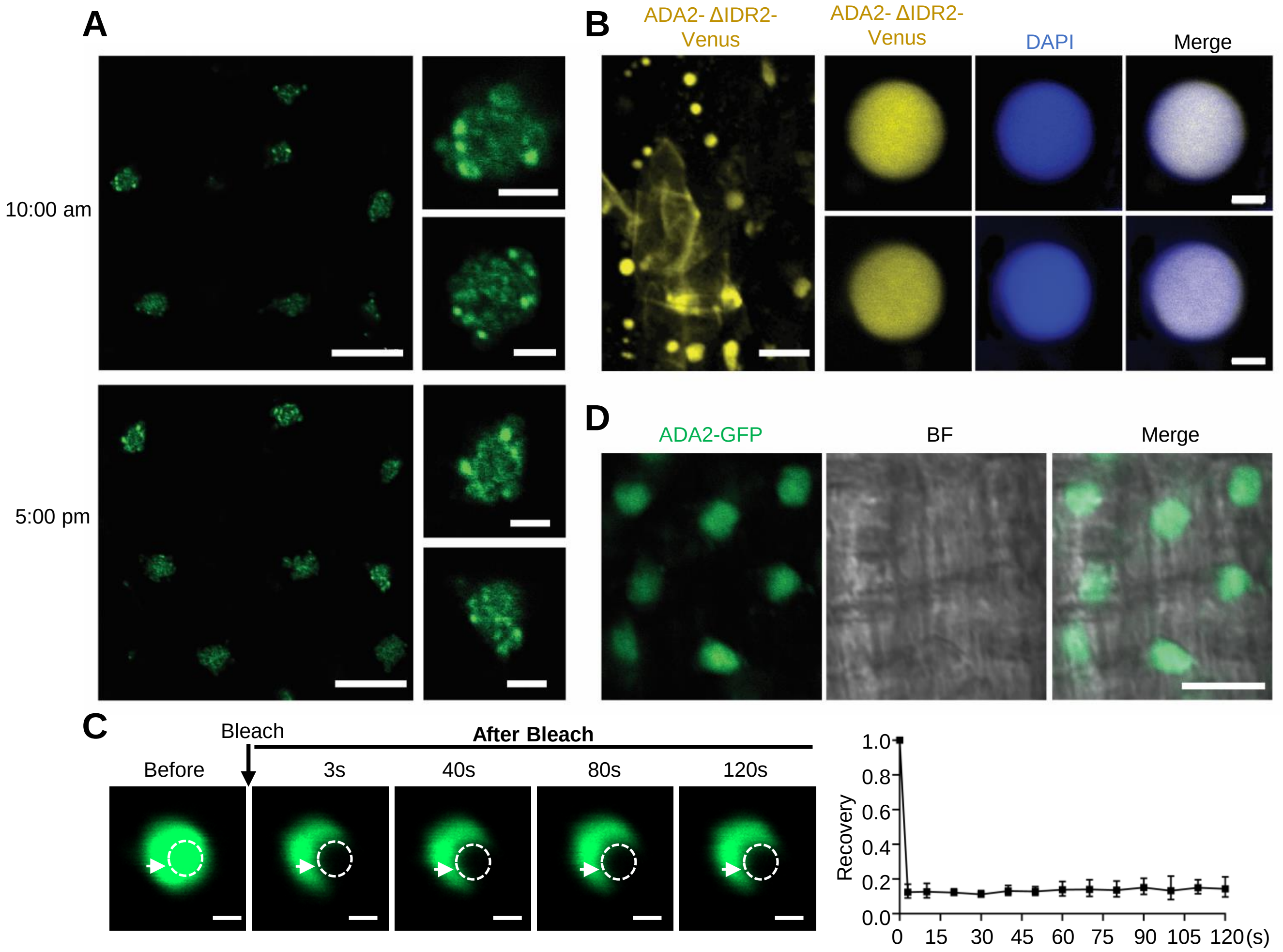


**Figure S2.** ADA2 forms condensates in the transgenic lines. A) Condensates were observed in the nuclei of *ADA2pro::ADA2-GFP* root tips at different time points. Bars=10 (left) and 2 (right) μm. B) ADA2 without IDR2 is diffusely distributed in the nucleus. Left panel, a fluorescence image indicating the overview of ADA2-ΔIDR2-Venus distribution in root tip cells. Right panel, enlarged nuclei stained with DAPI show the diffuse distribution of ADA2 without IDR2. Scale bars =10 μm (left) and 1 μm (right) , respectively. C) FRAP assay of ADA2 protein *in vivo.* Left panel. Representative images are shown. Bars = 1 μm. Right panel, quantification of relative fluorescence recovery of ADA2-GFP. Error bars represent the means ±SD from three independent biological replicates. D) ADA2-GFP was diffusely distributed in the nuclei within elongation zone. Bars=10 μm.

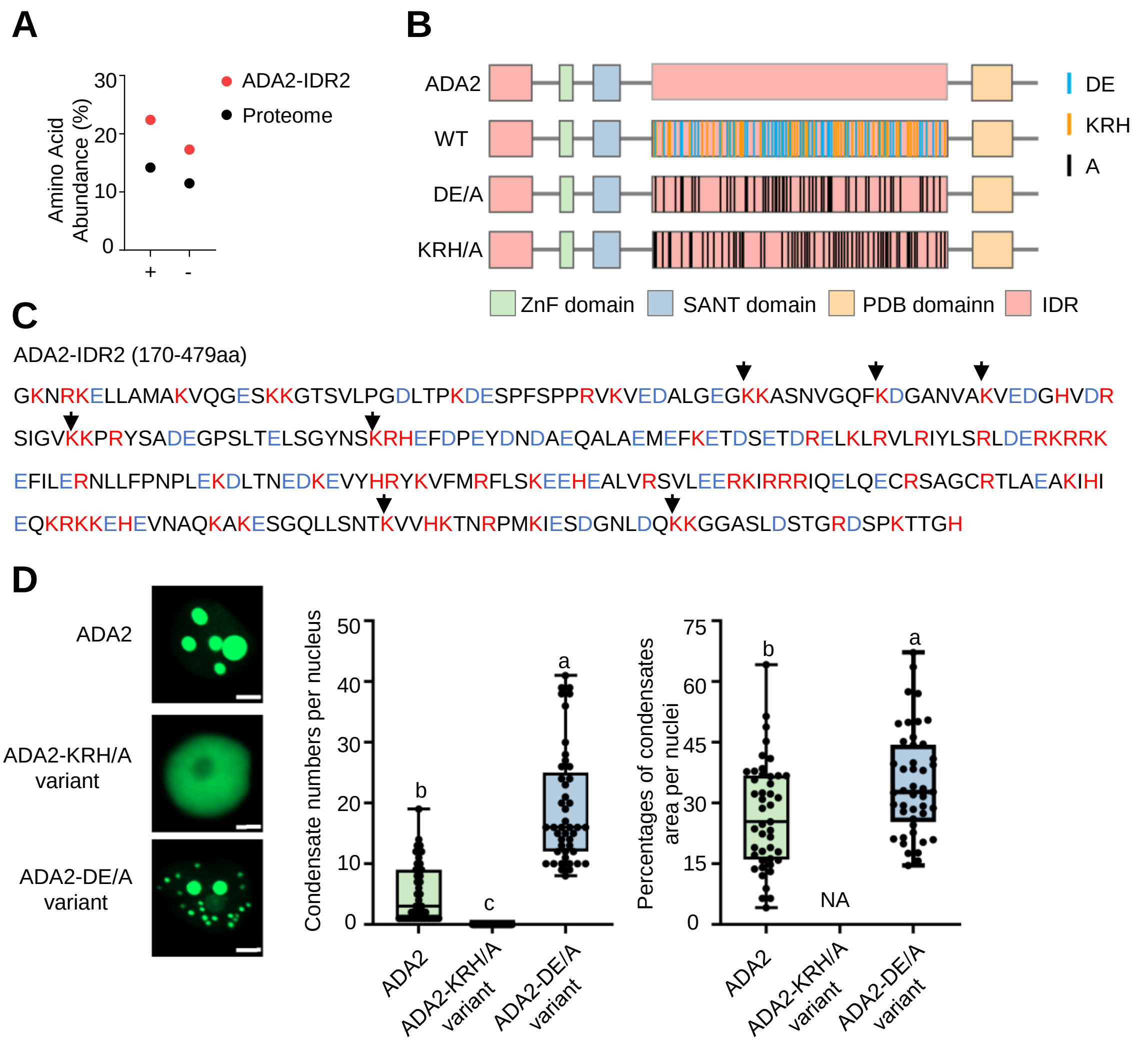


**Figure S3.** The polar charged amino acids of ADA2 determine protein aggregation *in vivo.* A) Abundance of polar charged amino acids in the sequences of ADA2 IDR2 and the proteome. B) Schematic diagrams indicating the distribution of positively (KRH) and negatively (DE) charged amino acid along the ADA2 IDR2 region. DE/A, the ADA2 variant with all D and E residues within IDR2 replaced by A. KRH/A, the ADA2 variant with all K, R, and H residues within IDR2 replaced by A. C) Protein sequence of the IDR2 of ADA2. Red and blue mark the positively charged and negatively charged amino acids, respectively. Arrows indicate the previously identified residues with acetylation modification. D) Left panel, images showing the nuclear distributions of ADA2-GFP, ADA2-KRH/A variant-GFP, and ADA2-DE/A variant-GFP proteins in the tobacco system. Bars = 5 μm. Right panel, statistics depicting the numbers and area percentages of condensates within nuclei. n = 45 (ADA2), 18 (ADA2-KRH/A variant), 45 (ADA2-DE/A variant). The boxplots shown the 25th and 75th percentiles (box), median and highest and lowest values. Error bars represent the means ±SD from independent biological replicates. The different significances were calculated using one-way ANOVA with Tukey's multiple comparison tests. Different letters on top of the bars indicate a significant difference (p-value < 0.05), and the same letters on top of bars indicate no significant difference.

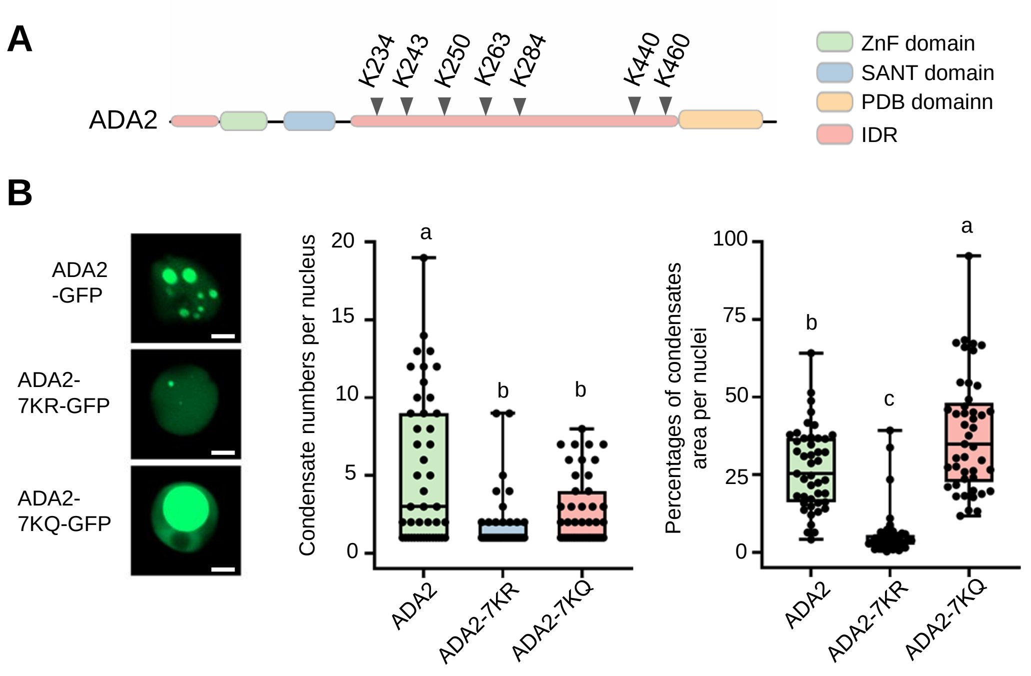


**Figure S4.** The acetylation of ADA2 influences the size of condensates. A) A schematic diagram shows the ADA2 protein structure, with the reported acetylated lysine residues indicated by arrowheads. B) Typical images show aggregation status of the indicated proteins in nuclei of tobacco cells. ADA2-7KR and ADA2-7KQ, are ADA2 variants with mutations in the seven lysine residues, where acetylation is replaced by either arginine or glutamine. Bars = 5 µm. Statistical data show the number and area percentages of condensates within nuclei (n=45 for each). The boxplots shown the 25th and 75th percentiles (box), median and highest and lowest values. Error bars represent the means ±SD from independent biological replicates as shown above. The different significances were calculated using one-way ANOVA with Tukey's multiple comparison tests. Different letters on top of the bars indicate a significant difference (p-value < 0.05), and the same letters on top of bars indicate no significant difference. The data for ADA2-GFP in Fig. S4B is copied from Fig. S3D. All the experimental groups in Fig. S4B and Fig. S3D were carried out within the same experiment.

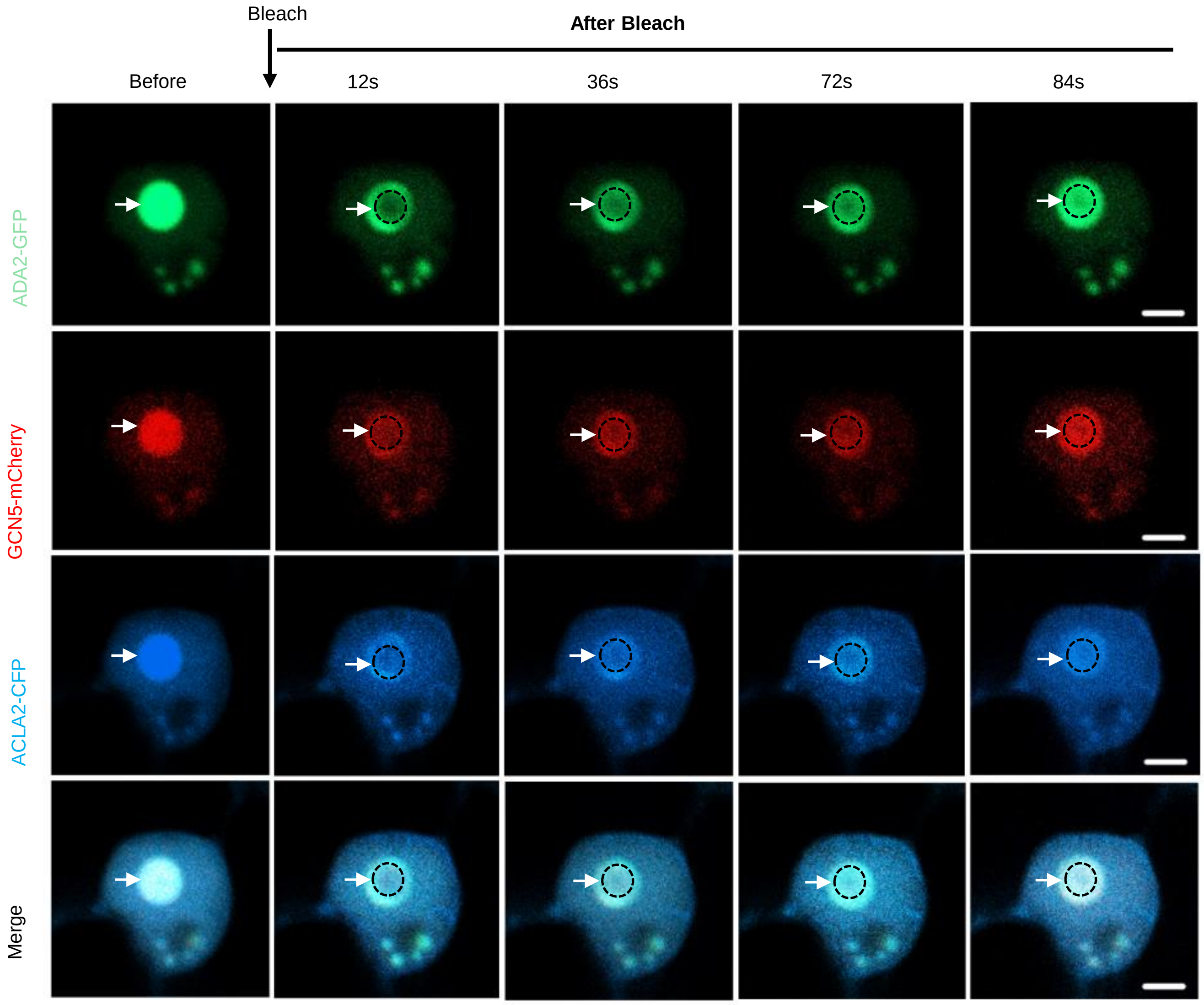


**Figure S5.** Fluorescence recovery after photobleaching (FRAP) assay for the GAA complex in tobacco system. Representative images before and after photobleaching for each channel are shown. Bars = 2 μm.

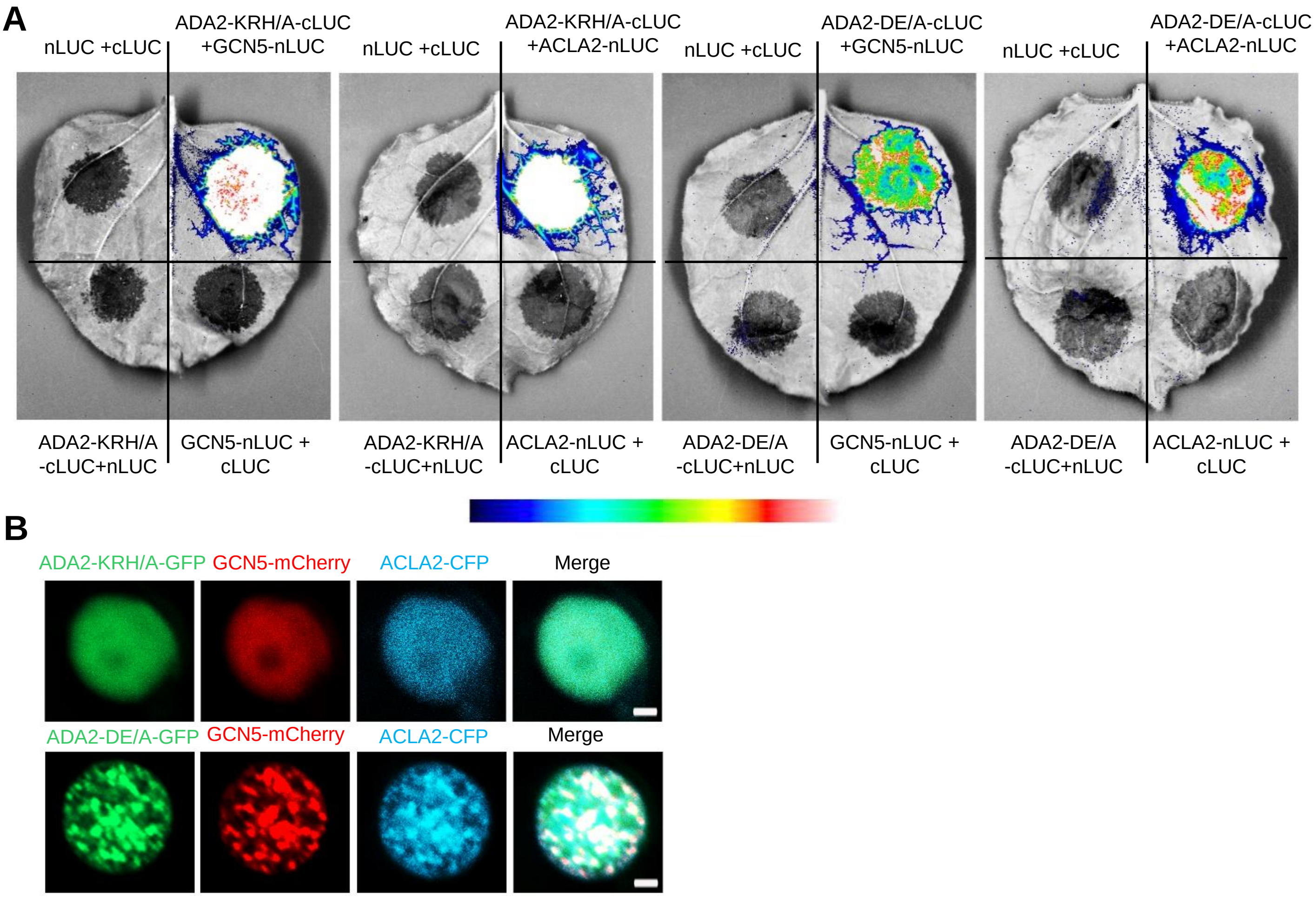


**Figure S6.** ADA2-KRH/A abolishes the phase separation of the GAA complex. A) Split-luciferase complementation assays (SLC) show the interaction between the indicated ADA2 variants and GCN5 or ACLA2 still exists in tobacco cells. B) Co-transformation of the ADA2 variants with GCN5 and ACLA2 in tobacco cells shows that the KRH/A mutation, but not the DE/A mutation, abolishes the phase separation of the GAA complex. Bars = 5 µm.

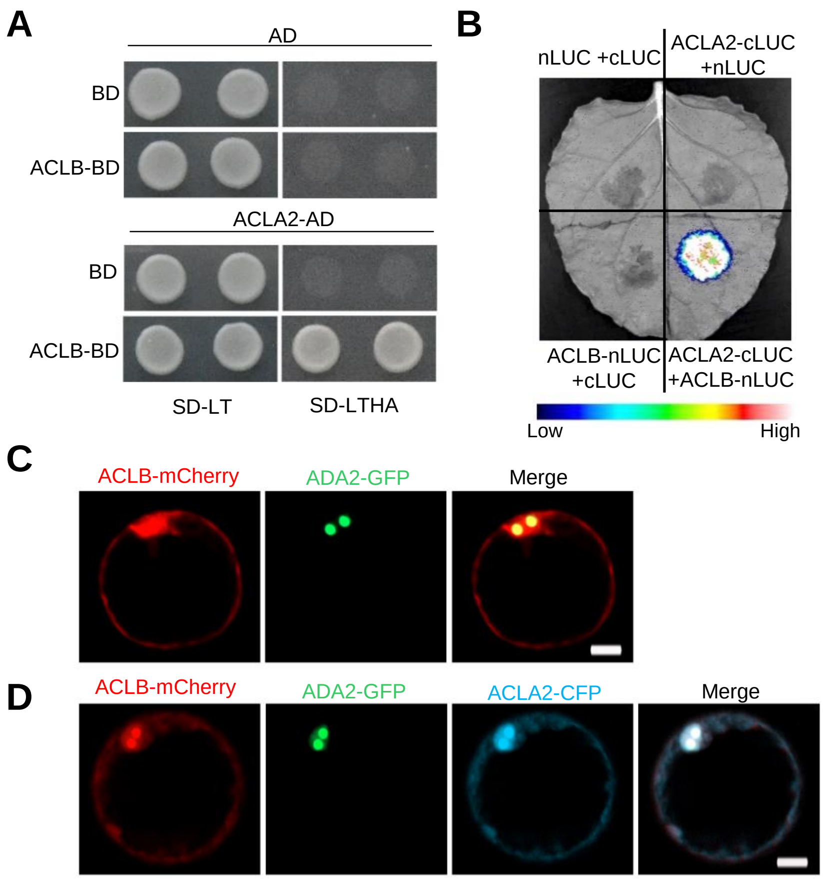


**Figure S7.** ACLA2 physically interacts with ACLB. A) Yeast two-hybrid assays of ACLA2 and ACLB in yeast cells. Full length cDNAs of *ACLB* and *ACLA2* were cloned into BD (the bait plasmid pGBKT7) and AD (the prey plasmid pGADT7), respectively. Yeast cells transformed with the indicated plasmid combinations were grown on control SD-LT medium and selective SD-LTHA medium. B) BiFC analysis showing the interaction between ACLA2 and ACLB in tobacco cells. C) ACLB-mCherry failed to form condensates in the nucleus when co-transformed with ADA2-GFP in rice protoplasts, Bars = 5 µm. D) ACLB-mCherry formed condensates in the nucleus when co-transformed with ADA2-GFP and ACLA2-CFP in rice protoplasts, Bars = 5 µm.

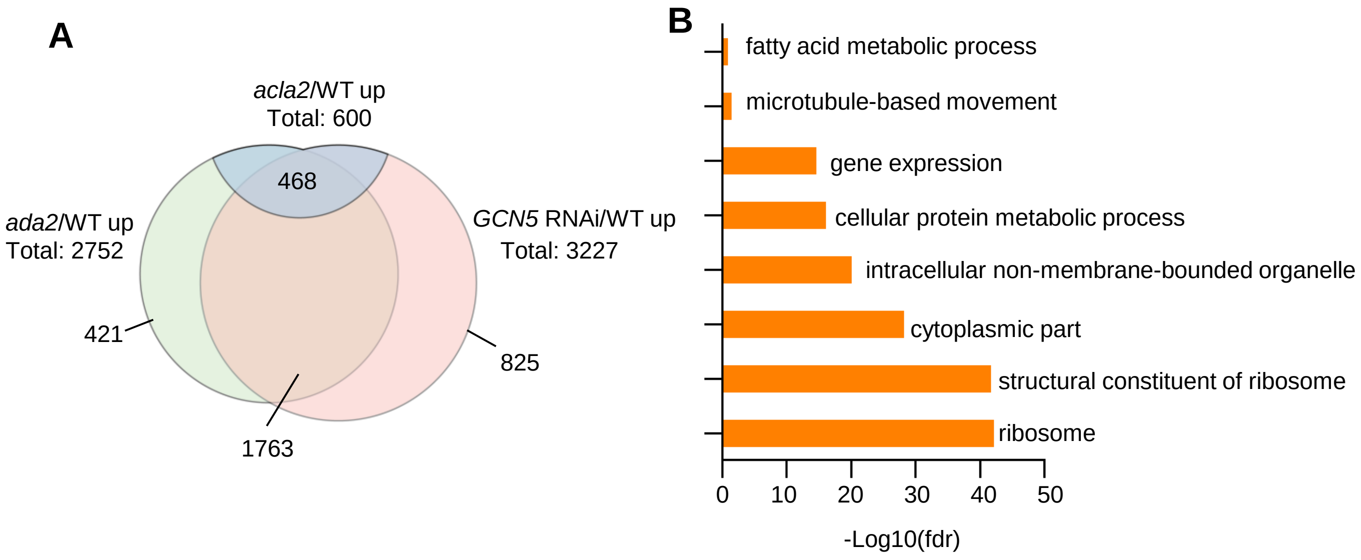


**Figure S8.** The upregulated gene assay of the GAA mutants. A) Overlap of upregulated genes in *GCN5* RNAi, *ada2,* and *acla2* compared to wild type. Fold changes are larger than 2 for *GCN5* RNAi and *ada2* and 1.5 for *acla2*, respectively, with *q*-value<0.05. B) Gene ontology assay for the co-upregulated genes (n=468).

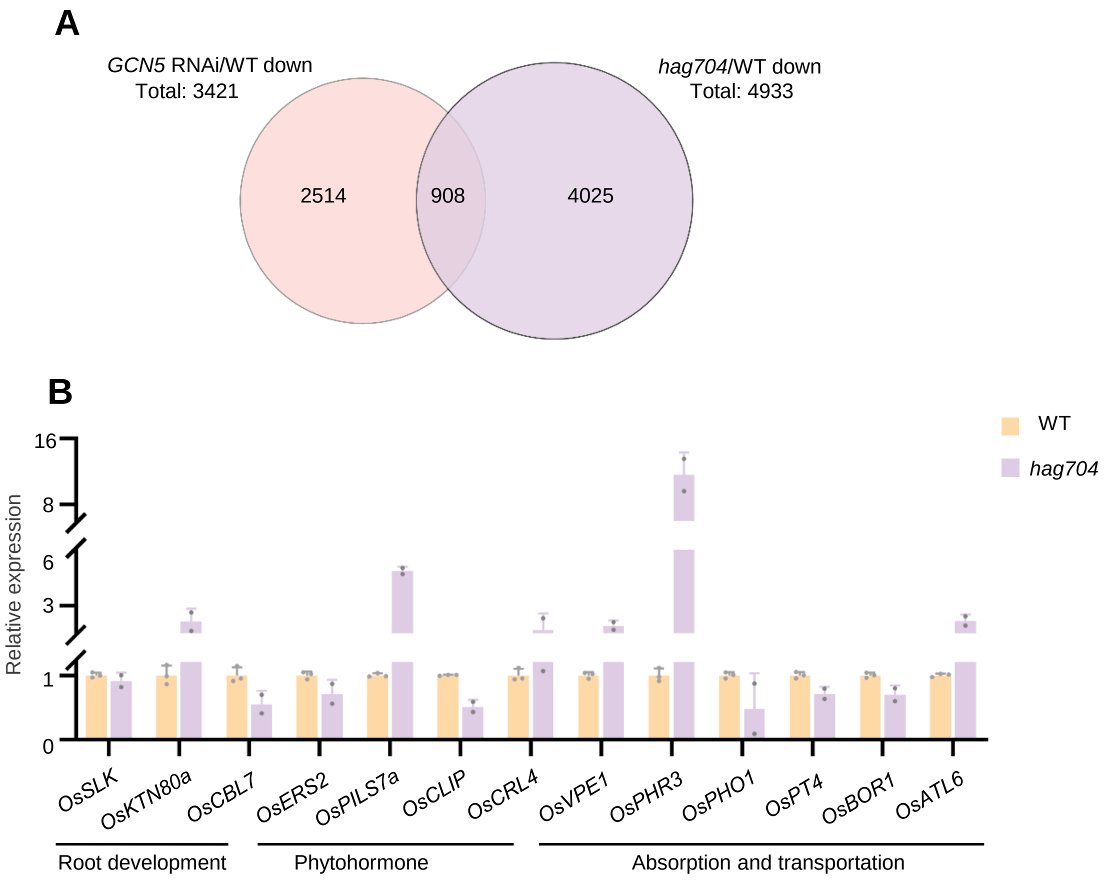


**Figure S9.** The comparison of downstream genes of GCN5 and HAG704. A) Overlap of downregulated genes in *GCN5* RNAi and *hag704* compared to wild type. Fold changes larger than 2 were used, with *q*-value<0.05. B) Histograms show transcription levels of the indicated key genes for root development. The values indicate the relative transcription levels compared to the wild type, with the wild type set as 1. n = 3 (WT), 2 (*hag704*). Error bars represent the means ± SD of biological replicates.

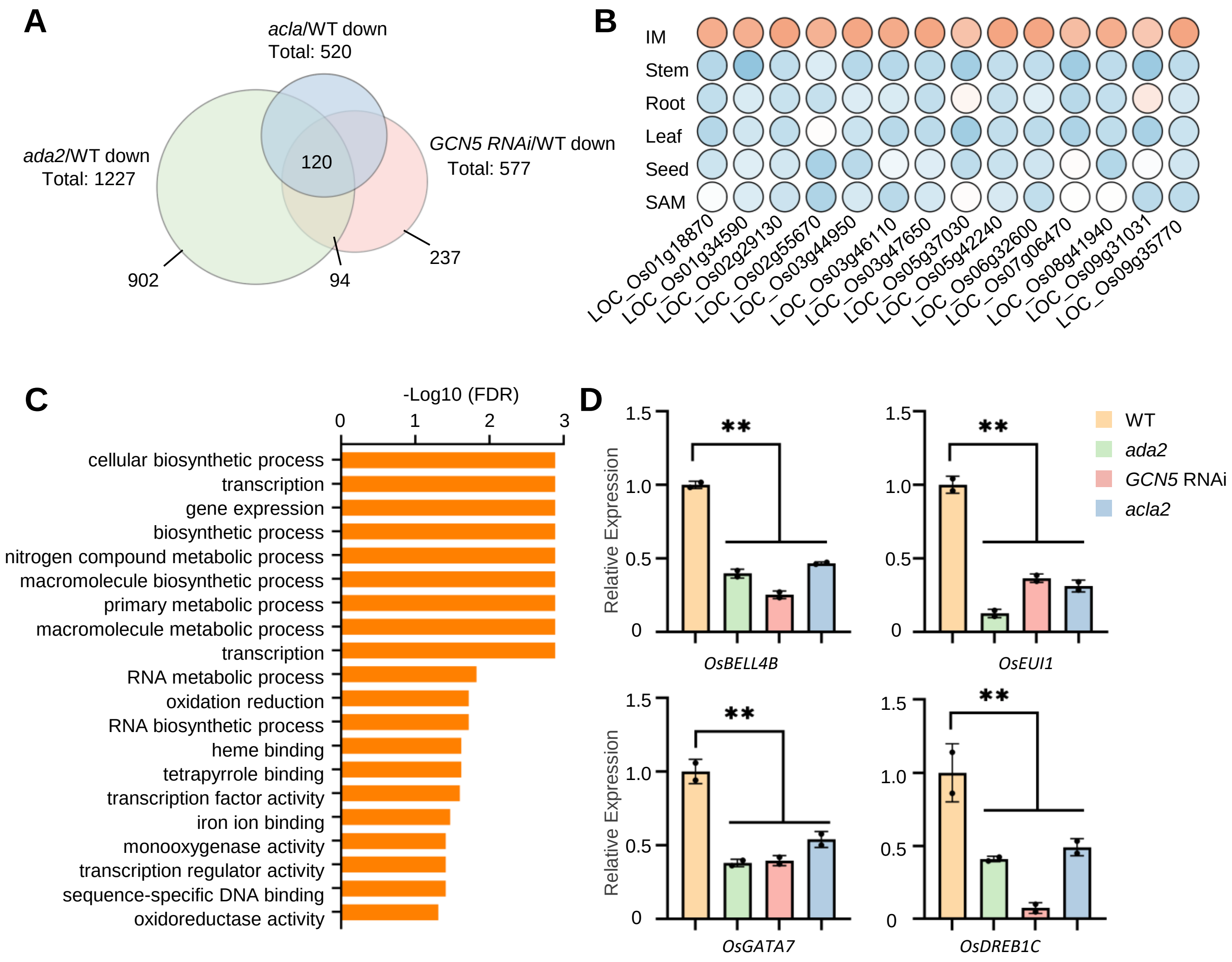


**Figure S10.** The GAA complex impacts gene transcription in inflorescence meristems. A) Overlap of down-regulated genes in *acla2*, *ada2* and *GCN5* RNAi. Fold changes are larger than 2 (GCN5 RNAi and ada2) and 1.5 (acla2), respectively, with *q*-value<0.05. B) Heat maps showing transcription profiles of genes specifically expressed at inflorescence meristems (IM). SAM, Shoot apical meristem. C) Gene ontology assay for the genes (n=120) that were downregulated in *GCN5 RNAi*, *ada2,* and *acla2* relative to the wild type. D) Histograms show genes transcription levels in the indicated background. The values indicate the relative transcription levels compared to the wild type, with the wild type set as 1. Error bars represent the means ± SD from two independent biological replicates. The different significances were calculated using one-way ANOVA with Tukey's multiple comparison tests. ** on top of the bars indicate a significant difference (p-value < 0.05).

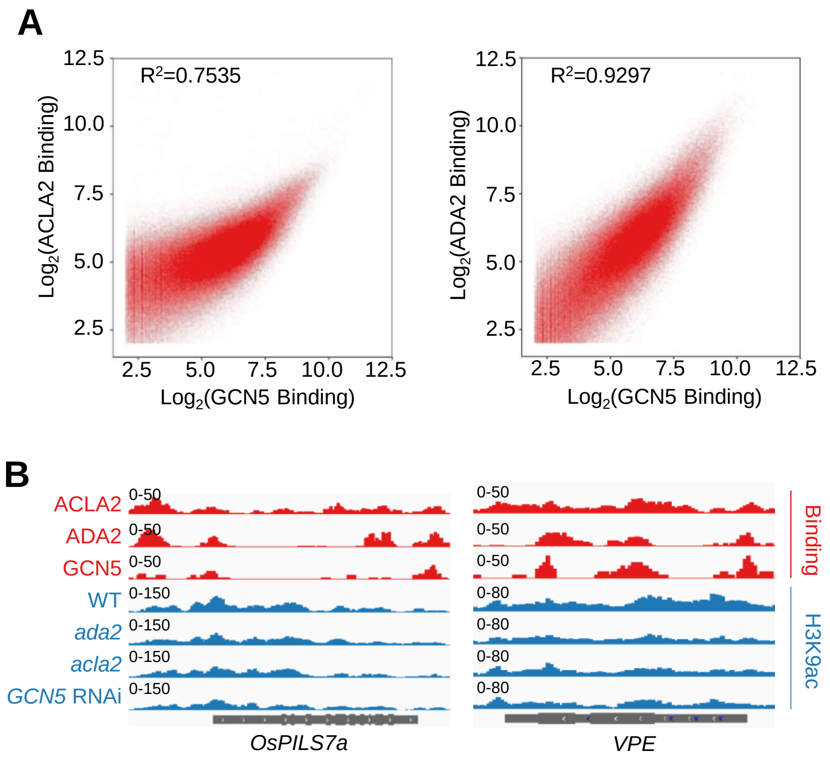


C

| MOTIF | TF |
|---|---|
| 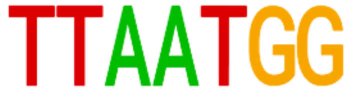 | OsWOX11 |
| 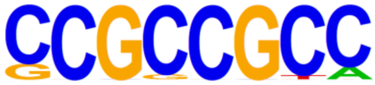 | AtSHN3 |
| 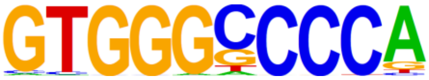 | ZNF692 |
| 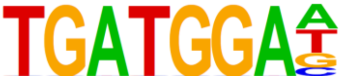 | HAP3 |

**Figure S11.** GCN5, ADA2, and ACLA2 jointly affect genomic H3K9 acetylation. A) Scatterplots showing the correlation of genomic binding strength between the two comparisons. The squared Pearson correlation coefficients ($R^2$) between the two variables are shown. B) Additional IGV screenshots show the binding signals for the indicated GAA components and the H3K9ac modification status in the specified backgrounds. C) Table showing the representatively enriched motifs within the GAA binding regions.

**A**

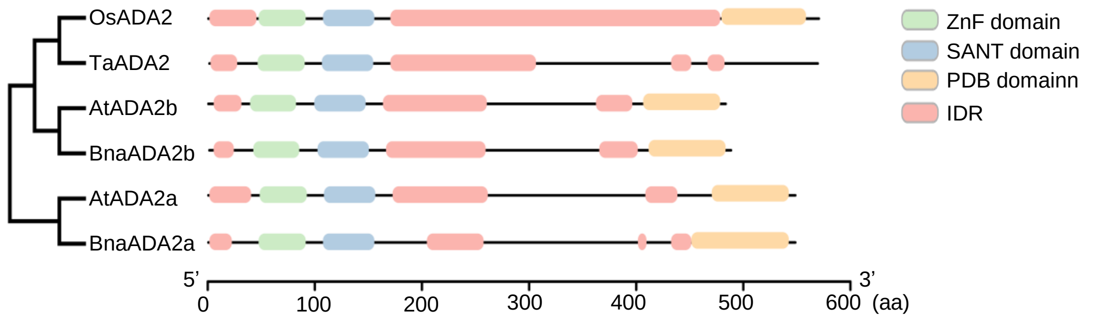


**B**

| Protein | The lenght of IDR (aa) | The number of KRH in IDR | Percent of KRH in IDR (%) | Percent of KRH in proteome (%) |
|---|---|---|---|---|
| AtADA2a | 106 | 25 | 23.58 | 11.65 |
| AtADA2b | 261 | 54 | 20.69 | 11.65 |
| BnaADA2a | 164 | 33 | 20.12 | 11.00 |
| BnaADA2b | 259 | 56 | 21.62 | 11.00 |
| TaADA2 | 206 | 41 | 19.90 | 10.92 |
| OsADA2 | 356 | 78 | 21.91 | 14.20 |

**C**

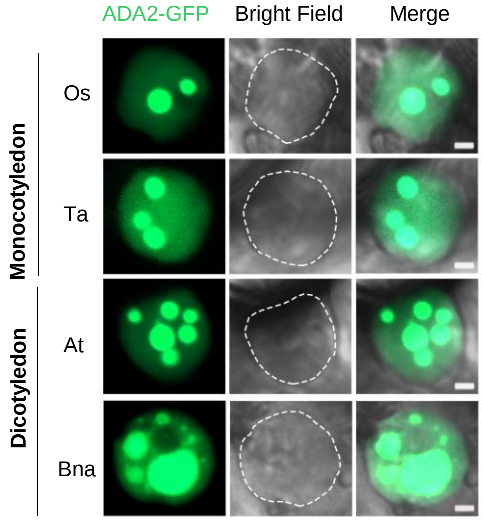


**Figure S12.** ADA2 aggregation in the nucleus is conserved across species. A) Phylogenetic tree of representative ADA2 proteins from various plant species including *Arabidopsis thaliana* (At), *Oryza sativa* (Os), *Brassica napus* (Bna), and *Triticum aestivum* (Ta). The scale bar represents the length of proteins**.** B) A table summarizing the characteristics of ADA2 from different species. C) ADA2 proteins from different species aggregate in the nucleus of tobacco cells. Bars = 2 μm.

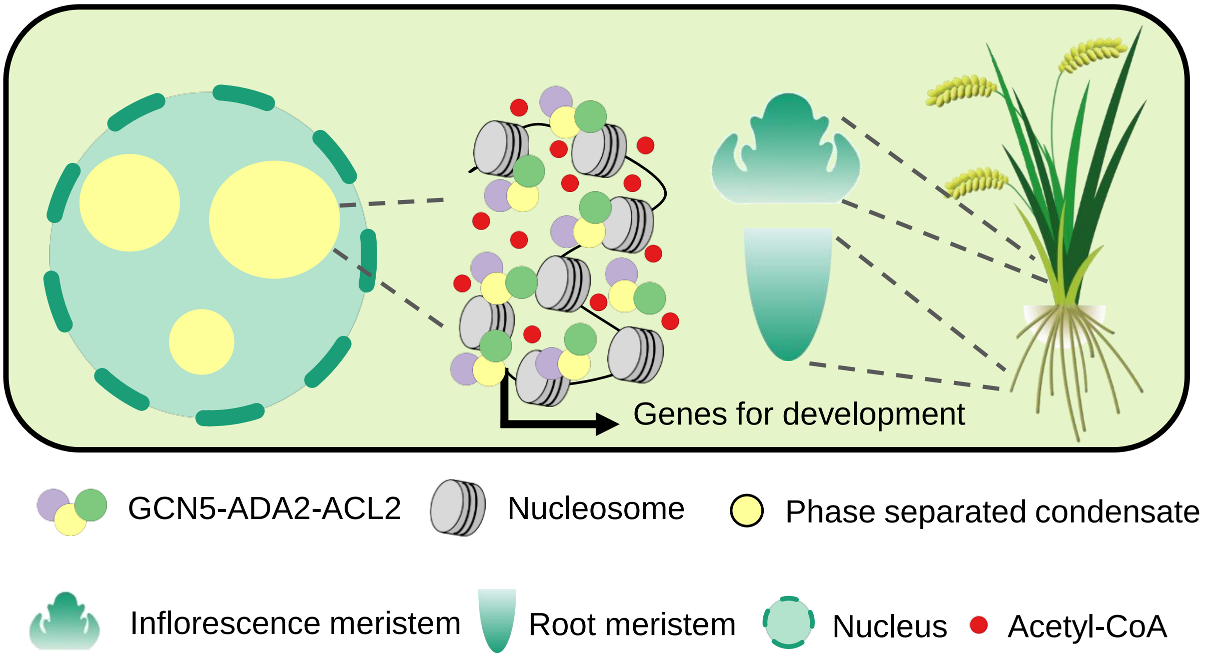


**Figure S13.** A proposed working model deciphering the function of the GAA complex in regulating root and inflorescence meristem development. In rice, GCN5-ADA2 and ACLA2 form a GAA complex in cells. Within this complex, ADA2, which possesses an intrinsically disordered region enriched with positively charged amino residues, directs the condensation of the GAA complex, while ACL enriches the acetyl-CoA concentration by cracking citrate in the microenvironment. The complex binds to specific genomic regions to facilitate histone acetylation and the transcription of key genes for root and inflorescence meristems.
