# Supplementary material for: ADA2 Forms Nuclear Condensates with GCN5 and ATP‐Citrate Lyase (ACL) to Modulate H3K9 Acetylation at Genes Functioning in Rice Meristems
Source: Adv Sci (Weinh). 2025 Nov 12;13(5):e13169. doi: 10.1002/advs.202513169 (PMC12849889; doi:10.1002/advs.202513169)
Supplement: Supplementary file 2 — Supporting Information [file ADVS-13-e13169-s002.docx]

**Table S1. RNA-seq analysis data**

| **Sample** | **Tissue** | **Replicate** | **Clean Reads** | **Mapping Reads** | **Unique mapping Reads** | **Mapping rate** |
| --- | --- | --- | --- | --- | --- | --- |
| WT | Root tips | 1 | 68,719,200 | 63,599,619 | 58,283,128 | 92.55% |
|  |  | 2 | 76,013,032 | 70,623,708 | 64,900,474 | 92.91% |
|  |  | 3 | 74,950,846 | 68,954,778 | 62,852,666 | 92.00% |
| *ada2* | Root tips | 1 | 77,072,380 | 67,083,790 | 58,288,168 | 87.04% |
|  |  | 2 | 65,796,352 | 57,933,687 | 50,981,102 | 88.05% |
|  |  | 3 | 55,585,474 | 48,376,038 | 42,081,140 | 87.03% |
| *GCN5* RNAi | Root tips | 1 | 61,497,598 | 50,827,764 | 41,778,474 | 82.65% |
|  |  | 2 | 59,133,614 | 49,553,968 | 41,001,866 | 83.80% |
|  |  | 3 | 75,761,876 | 65,079,451 | 54,873,784 | 85.90% |
| *acla2* | Root tips | 1 | 62,996,670 | 58,939,680 | 54,775,738 | 93.56% |
|  |  | 2 | 48,489,862 | 45,163,457 | 41,783,888 | 93.14% |
|  |  | 3 | 55,069,778 | 50,251,172 | 45,627,342 | 91.25% |
| *hag704* | Root tips | 1 | 71824898 | 62643733 | 58033110 | 87.22% |
|  |  | 2 | 116813596 | 104212634 | 98664822 | 89.21% |
| WT | Inflorescence meristem | 1 | 75,578,198 | 73,967,057 | 70,857,424 | 97.87% |
|  |  | 2 | 56,638,856 | 55,098,647 | 52,332,856 | 97.28% |
| *ada2* | Inflorescence meristem | 1 | 63,534,788 | 62,289,589 | 57,780,546 | 98.04% |
|  |  | 2 | 46,889,724 | 45,442,317 | 35,439,074 | 96.91% |
| *GCN5* RNAi | Inflorescence meristem | 1 | 45,993,778 | 45,122,994 | 43,476,594 | 98.11% |
|  |  | 2 | 49,165,398 | 48,246,741 | 44,865,738 | 98.13% |
| *acla2* | Inflorescence meristem | 1 | 65,185,176 | 63,957,628 | 59,983,490 | 98.12% |
|  |  | 2 | 51,251,694 | 50,315,576 | 47,124,066 | 98.17% |
