# Supplementary material for: ADA2 Forms Nuclear Condensates with GCN5 and ATP‐Citrate Lyase (ACL) to Modulate H3K9 Acetylation at Genes Functioning in Rice Meristems
Source: Adv Sci (Weinh). 2025 Nov 12;13(5):e13169. doi: 10.1002/advs.202513169 (PMC12849889; doi:10.1002/advs.202513169)
Supplement: Supplementary file 3 — Supporting Information [file ADVS-13-e13169-s001.docx]

**Table S2. CUT&Tag analysis data**

| **sample** | **replicate** | **antibody** | **clean Reads** | **mapping Reads** | **Mapping rate** |
| --- | --- | --- | --- | --- | --- |
| WT | 1 | H3K9ac | 54,476,600 | 45,840,086 | 84.15% |
|  | 2 | H3K9ac | 109,078,330 | 91,608,767 | 83.98% |
| *acla2* | 1 | H3K9ac | 88,024,472 | 81,737,325 | 92.86% |
|  | 2 | H3K9ac | 65,361,630 | 61,191,215 | 93.62% |
| *ada2* | 1 | H3K9ac | 31,619,352 | 30,524,981 | 96.54% |
|  | 2 | H3K9ac | 75,351,476 | 72,150,955 | 95.75% |
| *GCN5* RNAi | 1 | H3K9ac | 71,009,810 | 58,059,246 | 81.76% |
|  | 2 | H3K9ac | 143,176,846 | 116,751,392 | 81.54% |
| *ADA2pro::ADA2-GFP* | 1 | GFP | 36,343,650 | 32,175,554 | 88.53% |
|  | 2 | GFP | 85,995,646 | 76,149,760 | 88.55% |
| *GCN5pro::mcherry-GCN5* | 1 | mcherry | 22,652,684 | 4,659,079 | 20.57% |
|  | 2 | mcherry | 47,454,964 | 10,507,795 | 22.14% |
| *Ubipro::ACLA2-FLAG* | 1 | Flag | 45,022,274 | 38,804,533 | 86.19% |
|  | 2 | Flag | 51,172,982 | 43,718,446 | 85.43% |
| *35S::ADA2-GFP* (protoplast) | 1 | H3K9ac | 68,680,948 | 36,569,162 | 53.24% |
|  | 2 | H3K9ac | 64,418,854 | 42,845,468 | 66.51% |
| *35S::ADA2 KRH/A* variant*-GFP* (protoplast) | 1 | H3K9ac | 74,113,560 | 59,774,834 | 80.65% |
|  | 2 | H3K9ac | 63,352,284 | 50,107,183 | 79.09% |
