# Supplementary material for: ADA2 Forms Nuclear Condensates with GCN5 and ATP‐Citrate Lyase (ACL) to Modulate H3K9 Acetylation at Genes Functioning in Rice Meristems
Source: Adv Sci (Weinh). 2025 Nov 12;13(5):e13169. doi: 10.1002/advs.202513169 (PMC12849889; doi:10.1002/advs.202513169)
Supplement: Supplementary file 4 — Supporting Information [file ADVS-13-e13169-s006.docx]

**Table S3. Gene information within this study**

| **Locus** | **Gene name** | **annotation** |
| --- | --- | --- |
| LOC_Os03g53960 | *ADA2* | Transcriptional adaptor protein ADA2, histone acetyltransferase adaptor protein |
| LOC_Os10g28040 | *GCN5* | Histone acetyltransferase HAG702 |
| LOC_Os12g37870 | *ACLA2* | Subunit A of the heteromeric ATP-citrate lyase |
| LOC_Os01g19450 | *ACLB* | Similar to ATP-citrate lyase subunit B. |
| LOC_Os06g03600 | *SLK* | SEUSS-LIKE protein |
| LOC_Os10g35200 | *OsKTN80a* | Katanin regulatory subunit P80a, microtubule stabilizer |
| LOC_Os02g18880 | *OsCBL7* | Calcineurin B-like protein 7 |
| LOC_Os05g06320 | *OsERS2* | Ethylene response sensor 2 |
| LOC_Os09g38130 | *OsPILS7a* | Auxin efflux transporter |
| LOC_Os07g34110 | *OsCLIP* | transporter family domains |
| LOC_Os12g26940 | *OsCRL4* | Receptor-like serine/threonine kinase, cytokinin signaling |
| LOC_Os04g46880 | *OsVPE1* | Vacuolar phosphate efflux transporter, Pi homeostasis |
| LOC_Os02g04640 | *OsPHR3* | Transcription factor, regulation of Pi signaling and homeostasis |
| LOC_Os01g02000 | *OsPHO1* | Phosphate (Pi) transporter |
| LOC_Os04g10750 | *OsPT4* | Pi transporter |
| LOC_Os12g37840 | *OsBOR1* | Boron (B) transporter |
| LOC_Os02g09810 | *OsATL6* | Rice amino acid transporter-like 6 |
| LOC_Os03g52239 | *OsBELL4B* | BEL1-like homeobox transcription factor |
| LOC_Os05g40384 | *OsEUI1* | Cytochrome P450 monooxygenase |
| LOC_Os06g03670 | *OsDREB1C* | Dehydration-responsive element binding protein 1C, |
| LOC_Os10g40810 | *OsGATA7* | GATA transcription factor |
