# Supplementary material for: ADA2 Forms Nuclear Condensates with GCN5 and ATP‐Citrate Lyase (ACL) to Modulate H3K9 Acetylation at Genes Functioning in Rice Meristems
Source: Adv Sci (Weinh). 2025 Nov 12;13(5):e13169. doi: 10.1002/advs.202513169 (PMC12849889; doi:10.1002/advs.202513169)
Supplement: Supplementary file 6 — Supporting Information [file ADVS-13-e13169-s005.pdf]

Figure 1 source data

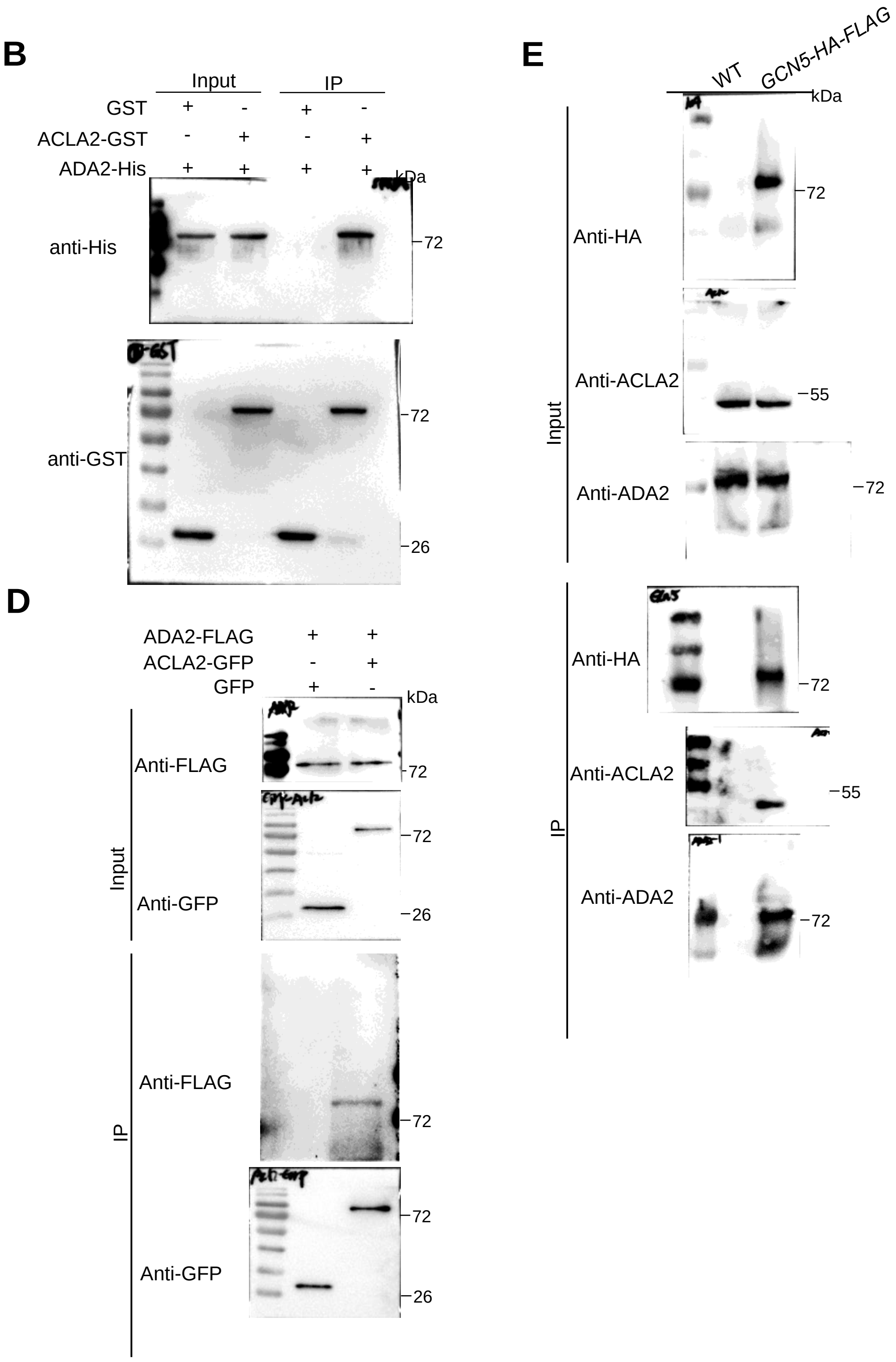

Figure 5 source data

C

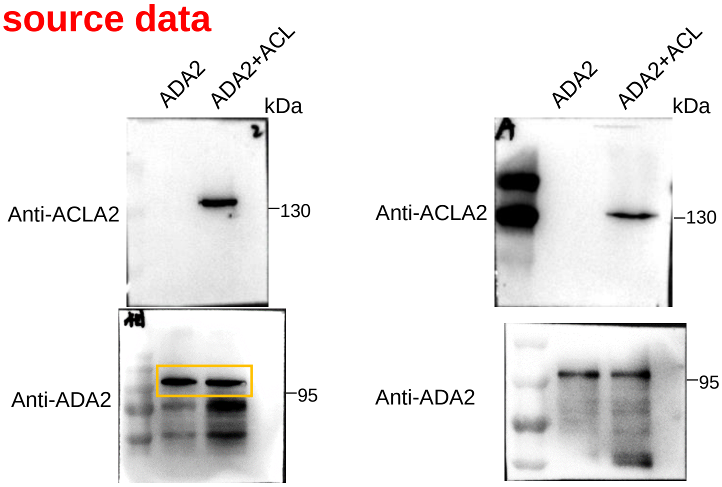

D

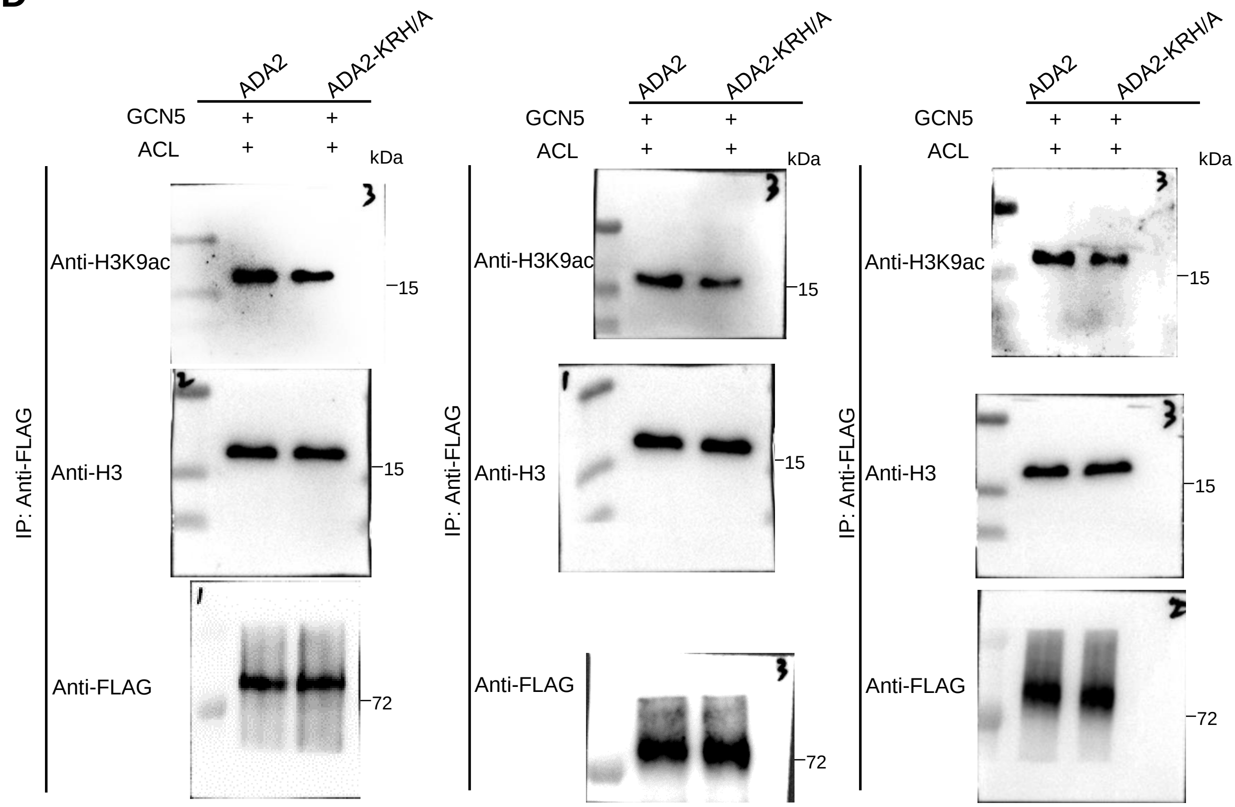

Figure S3D and Figure S4B source data (ADA2-GFP phase separation)

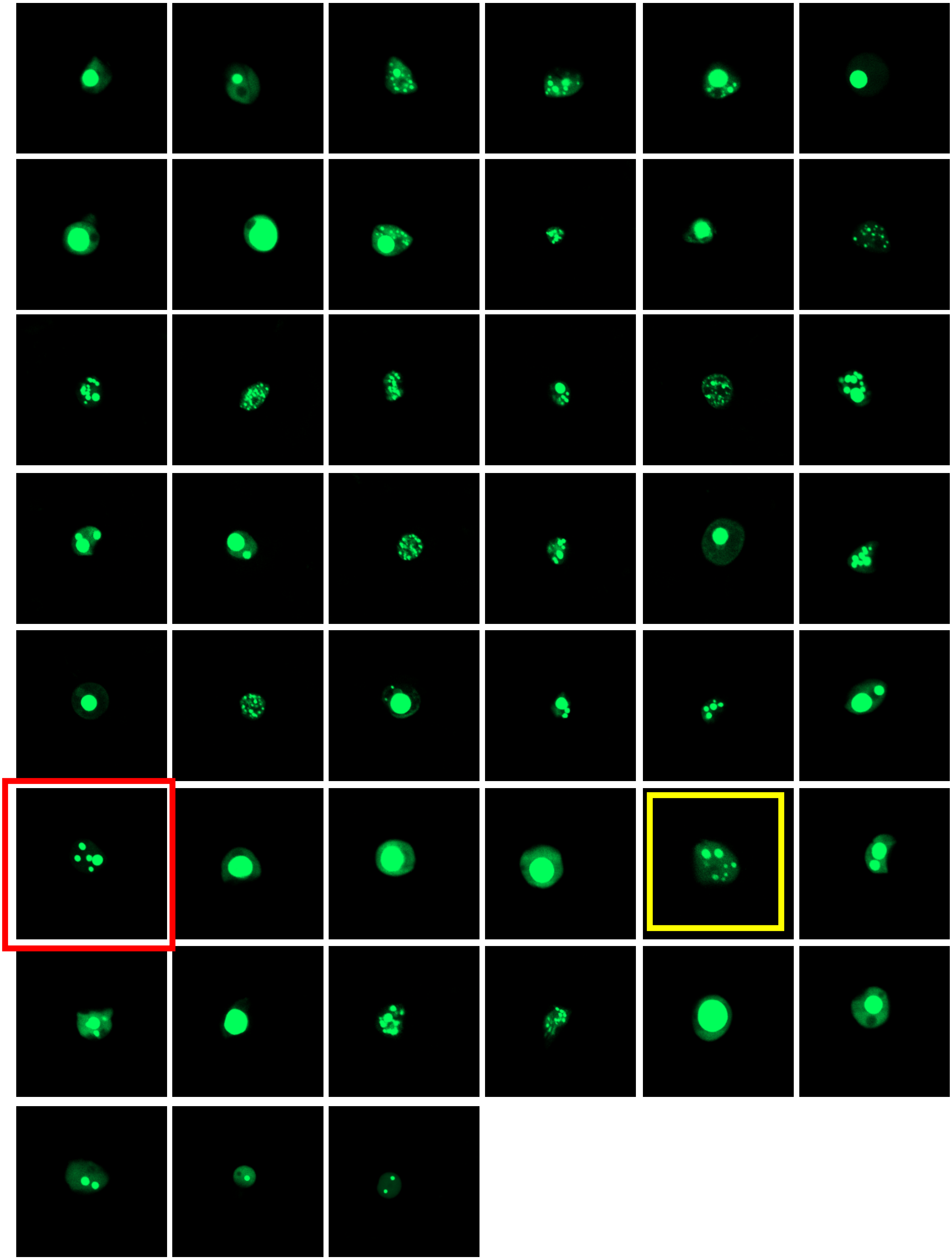

The images marked by the red and yellow boxes are used in this work.

Figure S3D source data (ADA2-KRH/A variant)

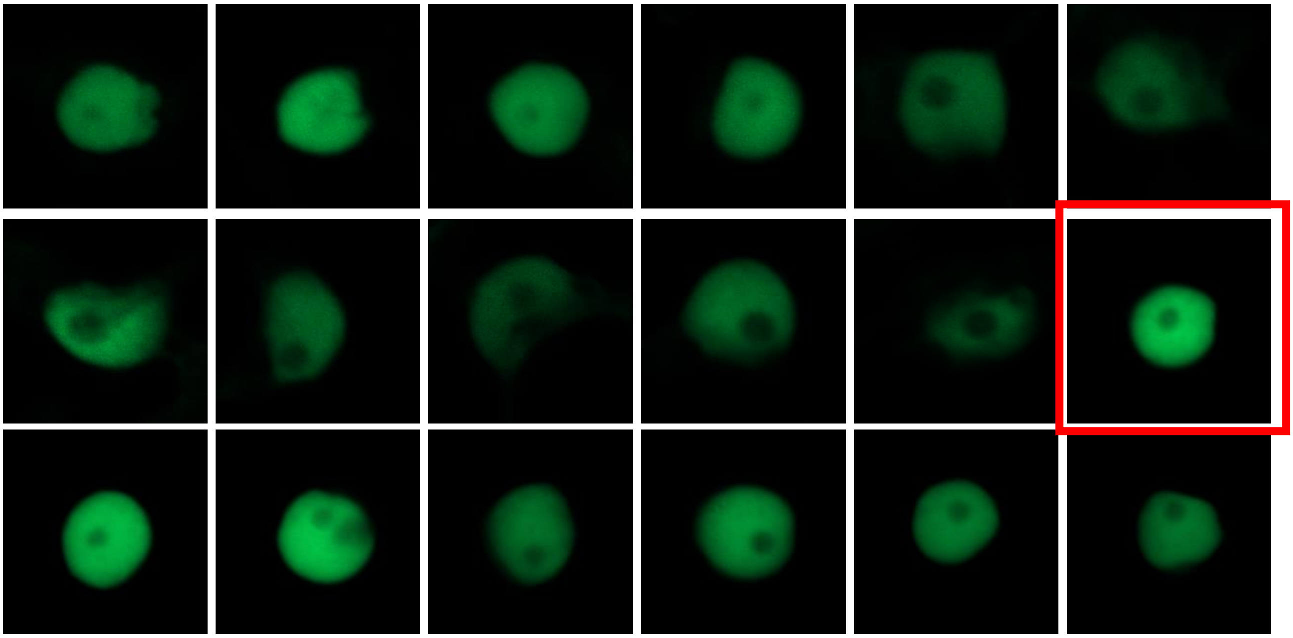

The image marked by the red box is used in this work.

Figure S3D source data (ADA2-DE/A variant)

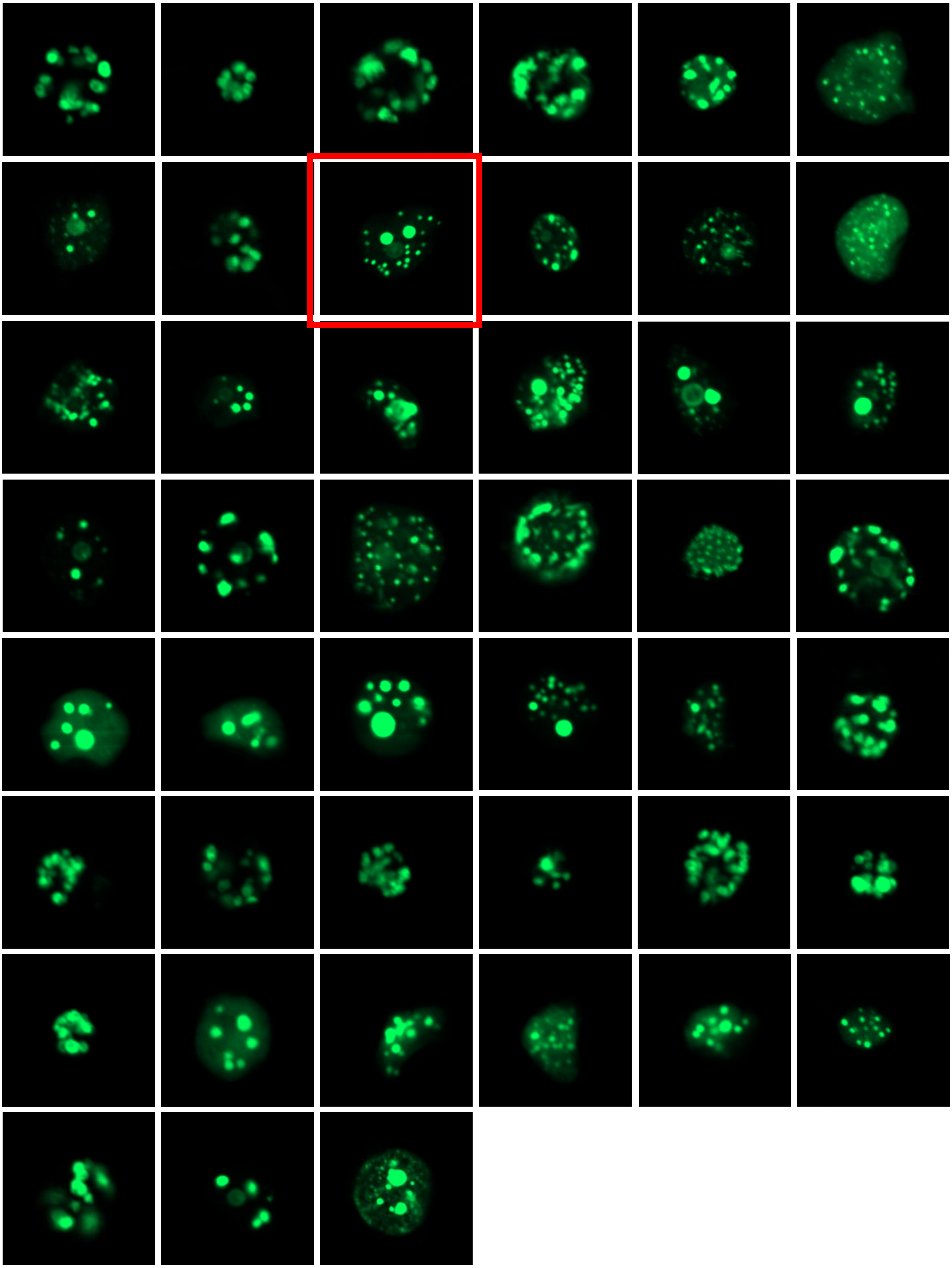

The image marked by the red box is used in this work.

Figure S4B source data (ADA2-7KR-GFP)

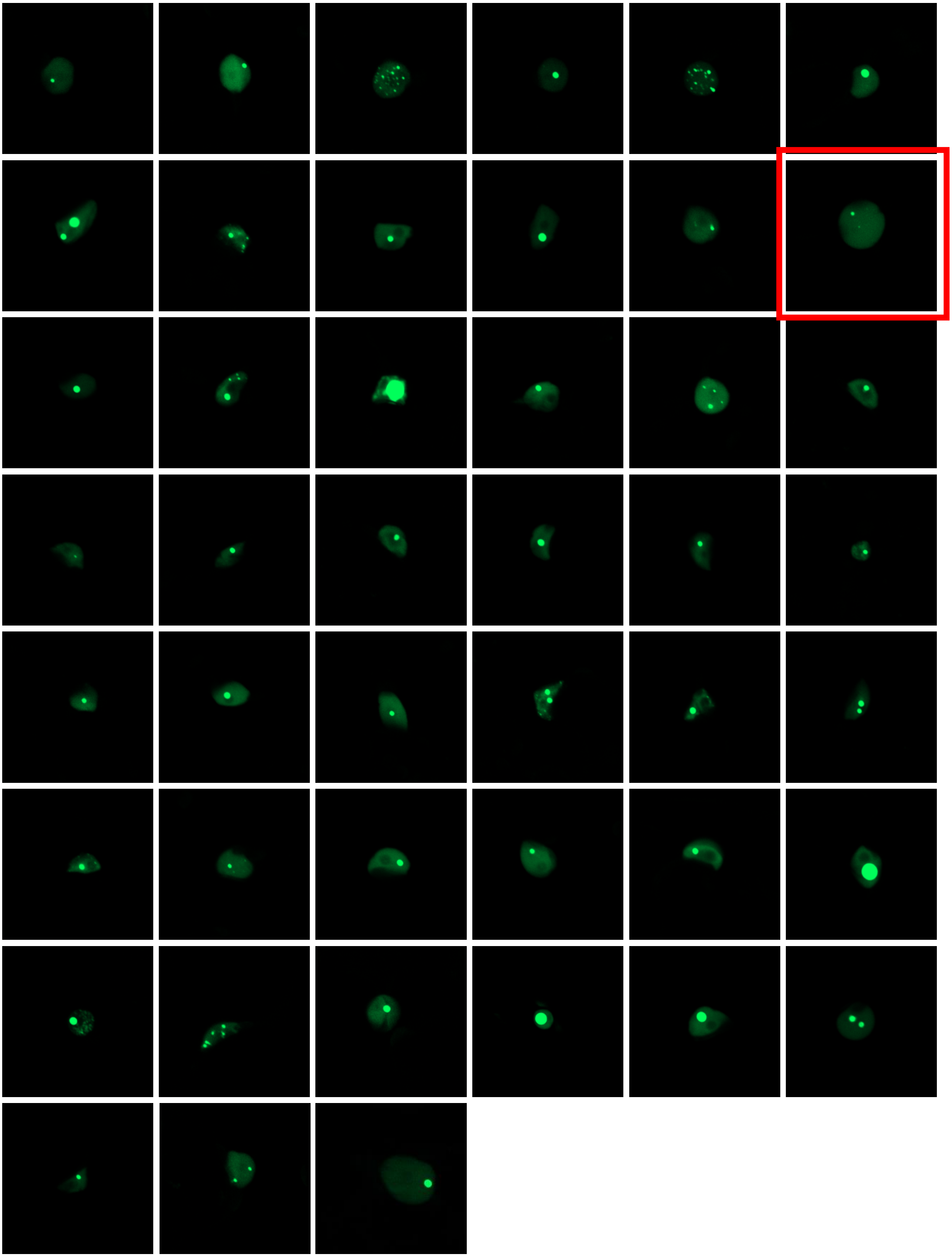

The image marked by the red box is used in this work.

Figure S4B source data (ADA2-7KQ-GFP)

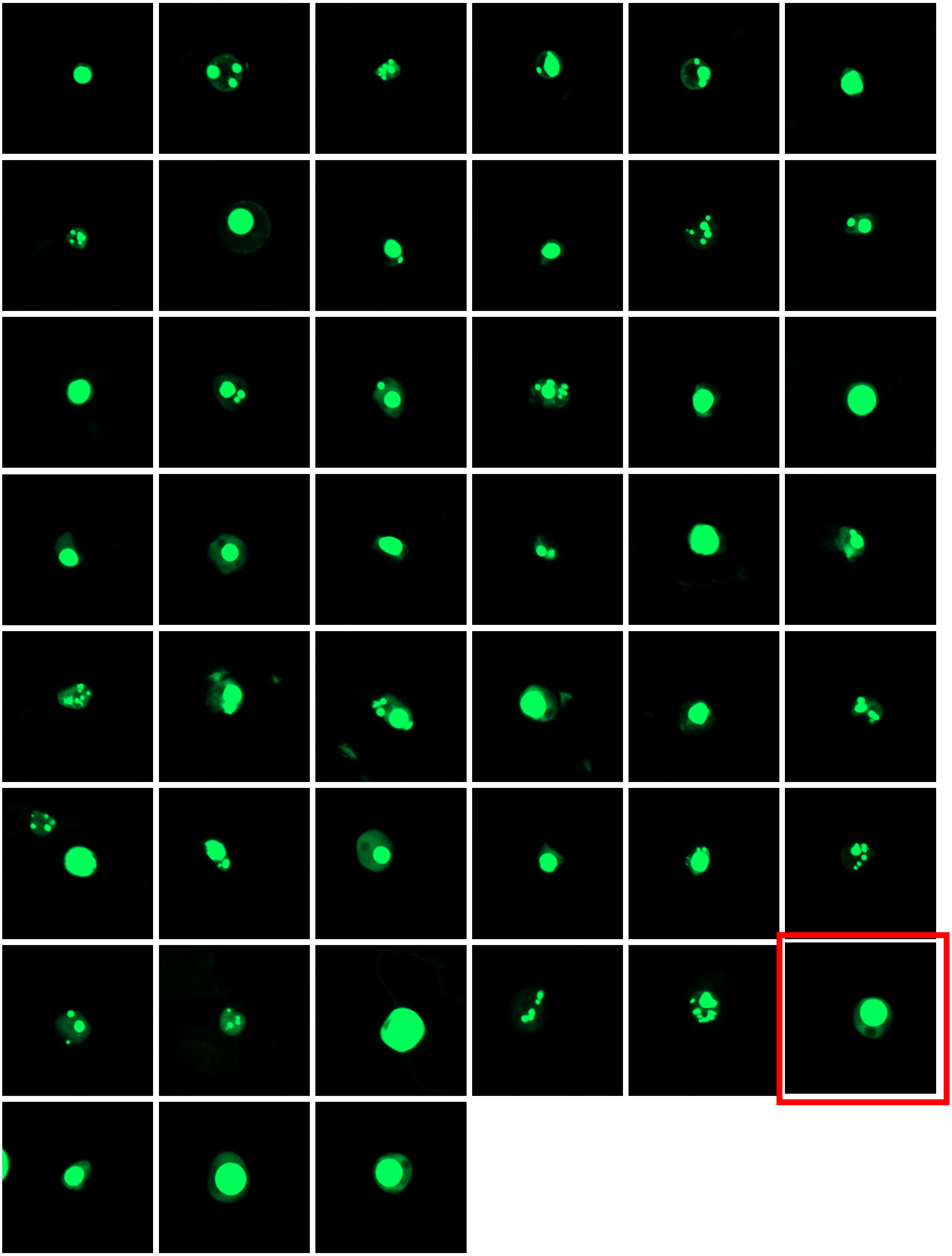

The image marked by the red box is used in this work.

Figure S12C source data

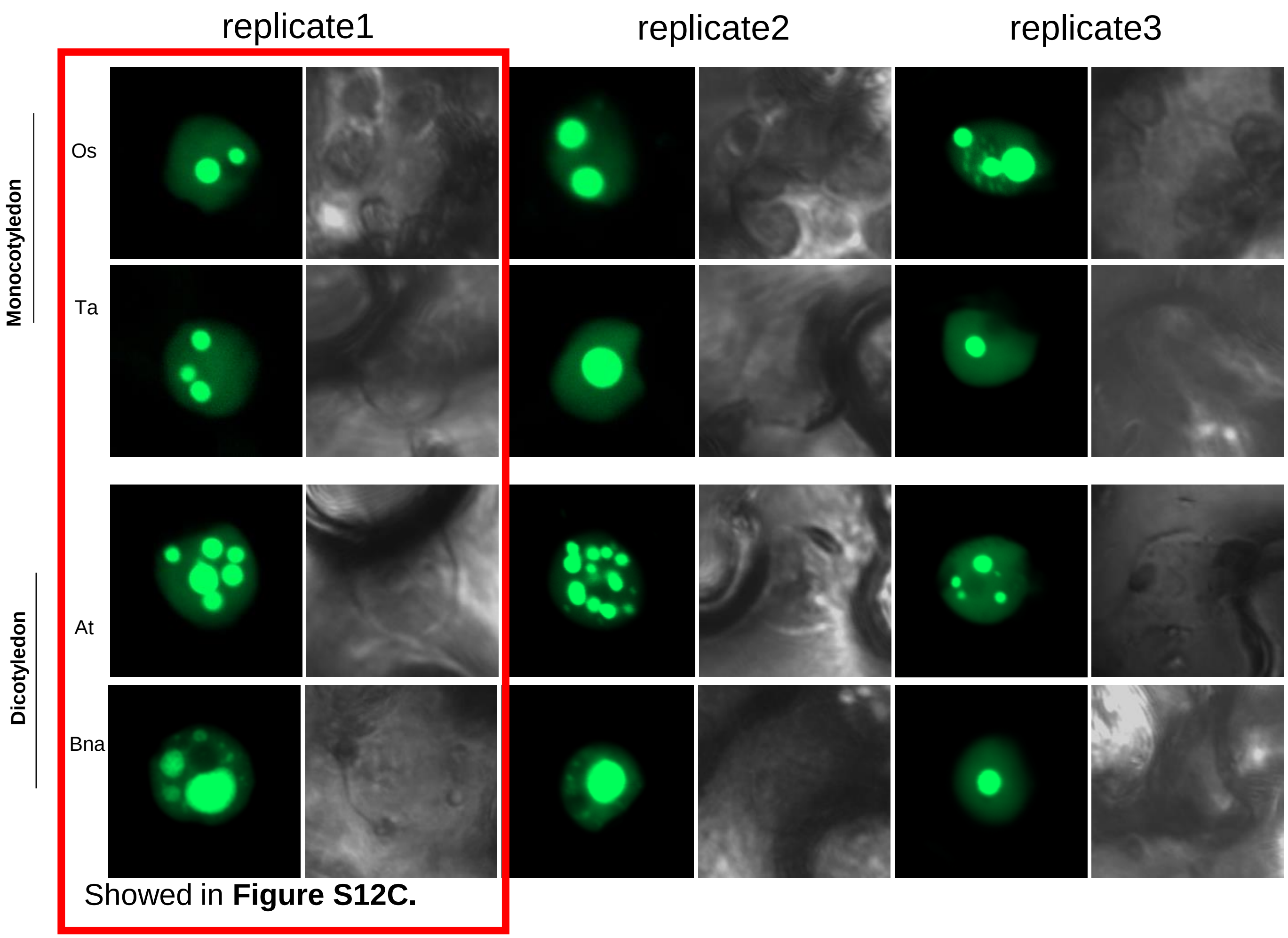

The images marked by the red boxes are used in this work.
